# Gaze-informed, task-situated representation of space in primate hippocampus during virtual navigation

**DOI:** 10.1371/journal.pbio.2001045

**Published:** 2017-02-27

**Authors:** Sylvia Wirth, Pierre Baraduc, Aurélie Planté, Serge Pinède, Jean-René Duhamel

**Affiliations:** 1 Centre de Neuroscience Cognitive, UMR 5229, CNRS and University of Lyon, Bron, France; 2 GIPSA-lab, UMR 5216, CNRS and University of Grenoble-Alpes, Saint Martin d'Hères, France; National Institute of Mental Health, United States of America

## Abstract

To elucidate how gaze informs the construction of mental space during wayfinding in visual species like primates, we jointly examined navigation behavior, visual exploration, and hippocampal activity as macaque monkeys searched a virtual reality maze for a reward. Cells sensitive to place also responded to one or more variables like head direction, point of gaze, or task context. Many cells fired at the sight (and in anticipation) of a single landmark in a viewpoint- or task-dependent manner, simultaneously encoding the animal’s logical situation within a set of actions leading to the goal. Overall, hippocampal activity was best fit by a fine-grained state space comprising current position, view, and action contexts. Our findings indicate that counterparts of rodent place cells in primates embody multidimensional, task-situated knowledge pertaining to the target of gaze, therein supporting self-awareness in the construction of space.

## Author summary

In the brain of mammalian species, the hippocampus is a key structure for episodic and spatial memory and is home to neurons coding a selective location in space (“place cells”). These neurons have been mostly investigated in the rat. However, species such as rodents and primates have access to different olfactory and visual information, and it is still unclear how their hippocampal cells compare. By analyzing hippocampal activity of nonhuman primates (rhesus macaques) while they searched a virtual environment for a reward, we show that space coding is more complex than a mere position or orientation selectivity. Rather, space is represented as a combination of visually derived information and task-related knowledge. Here, we uncover how this multidimensional representation emerges from gazing at the environment at key moments of the animal’s exploration of space. We show that neurons are active for precise positions and actions related to the landmarks gazed at by the animals. Neurons were even found to anticipate the appearance of landmarks, sometimes responding to a landmark that was not yet visible. Overall, the place fields of primate hippocampal neurons appear as the projection of a multidimensional memory onto physical space.

## Introduction

Place cells are the quintessential signature of hippocampal neural activity in rodents and code the animal’s position in an environment [1,2]. These neurons’ place selectivity and directionality strongly depend on the visual and/or vestibular cues, as has recently been shown in virtual reality settings in rodents [3–7]. Place cells are observed too in humans navigating virtual environments [8,9] and in other primates in real and virtual environments [10–12]. Yet, there is no consensus on how hippocampal place cells found in monkeys or humans precisely compare to place cells in rodents in the real or virtual world. Previous work [10,11] suggested that, unlike in rodents, space in the primate hippocampus may be coded in a gnostic, landmark-centered representation. Neurons in the monkey hippocampus were shown to convey much more information about the spatial view than about the place, eye position, or head direction. Although there was some modulation of spatial view responsiveness by place [13], the animal’s target of gaze (i.e., what the animal was looking at) was paramount in explaining firing rate [10]. These observations contradict other studies in macaques [14–16] and in humans in virtual reality mazes [8,9] describing robust place-coding activity. In the latter human single-cell studies [8,9], some cells were sensitive to the conjunction of place and goal or place and view, demonstrating complex task-related coding. However, as eye tracking was not feasible in these studies, neural activity was not analyzed with respect to eye movements and visual exploration. Thus, it remains unclear how active vision informs the neural construction of space at the single-cell level in the primate.

In the present study, we probed the nature of hippocampal coding in a goal-oriented task, separating goal and visual landmarks, and examined jointly how cells code for position, direction, and target of gaze. The goal-oriented setting enabled us to examine whether the task-related context of navigation modulated the activity of the cells. We thus analyzed firing in a discrete state space in which the animal’s trajectory in the maze is segmented into elemental transitions from one state in the environment to another [17–19]. Though our results are limited to virtual reality (VR), the recent use of this technique in humans [9] and in rodents [3–7,20] provides an apt comparison of the hippocampal coding in an environment in which spatial information is principally derived from visual input. In this framework, our results give a comprehensive and thorough analysis of the variables controlling the activity of hippocampal cells, bridging the gap between studies in rodents and in primates—including humans—collected in real and virtual environments. We show how hippocampal cells code for the target of gaze in an informed manner, embedding self-position with respect to elements in the environment and to action context. Thereby, we bring a new perspective on models of hippocampal spatial function by focusing on the role of the idiosyncratic visual exploration in primates in constructing a representation of the world that is highly useful to wayfinding.

## Results

### Animals use direction and compute trajectory in the virtual world

**Fig 1:**
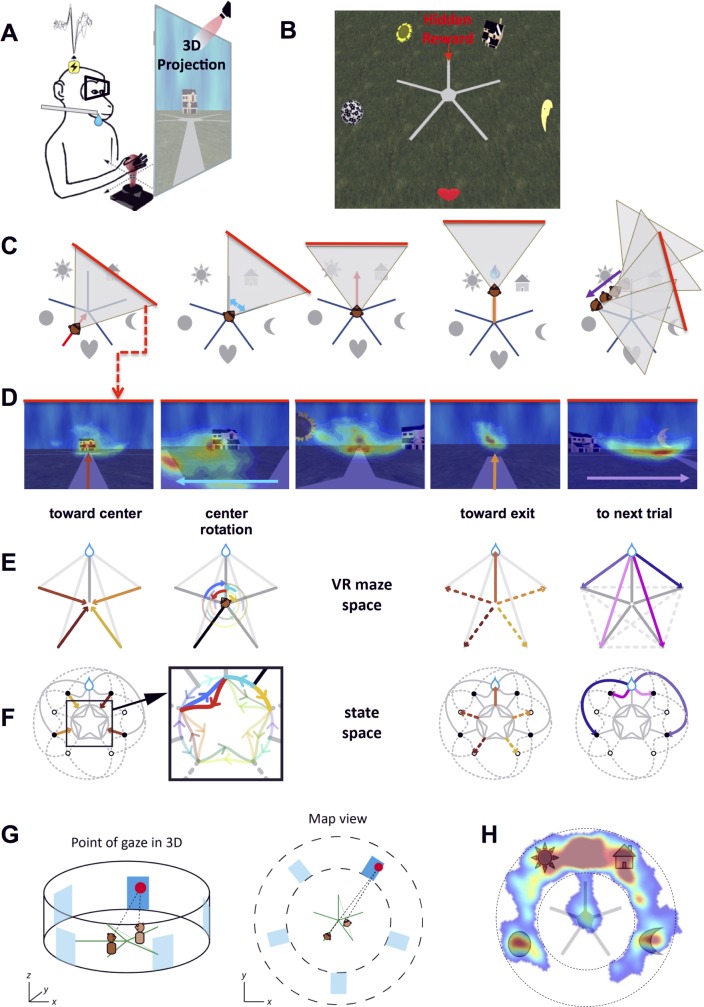
Experimental setup and behavioral task. **A.** Experimental setup. The animal was seated in front of a 152 x 114 cm screen on which a computer-generated scene was projected in stereo. The animal was equipped with shutter glasses synchronized with the projection and could move in the virtual world via a joystick. A juice dispenser delivered reward directly in the animal’s mouth when the monkey reached a hidden rewarded area. **B.** View from above of the star maze. Five landmarks were placed between the five arms of the maze at a radius twice the arms’ length. A reward was given to the animal when he reached the end of an arbitrarily chosen arm (in this case, the arm between the sunflower and the house). **C.** A sequential illustration of the animal’s position and field of view at key representative events of a trial. (1) The animal starts at one end of a path and moves towards the center, (2) turns left or right in the center, (3) chooses one path, (4) enters the chosen path and is rewarded at the end if correct, and (5) the animal is relocated (joystick disengaged) to the next start. **D.** First-person view of the five events described in **C**, with a heat map of the monkey’s gaze fixations overlaid on the scene illustrating the animal’s scanning interests. Arrows indicate the main direction of motion of the animal. **E.** Illustration of the steps described in **C** and **D** in the actual maze space. Monkey’s moves are represented by colored arrows. **F.** Illustration of the state space in which neuronal data was analyzed. The same steps as in **E** are plotted in the state-space graph with corresponding colors. For convenience, the animal’s current position in the graph also denotes the animal’s current straight ahead direction. For example, a position in the northeastern part of the graph corresponds to the animal viewing the northeast from its physical position. The state-space representation parses the animal’s trajectories into a series of action- or position-triggered transitions between choice points (graph vertices). Starting positions are figured as black dots. All actions that can eventually lead to the reward are in solid lines, while dashed lines indicate either erroneous actions leading to the end of unrewarded arms (open circles) or the path to the next start, outside the maze arms. This representation allows describing in a similar way the moves that include a translation and the purely rotational moves made in the center of the maze (expanded inset in the black square). Rotations of 72° (angle between two maze arms) are mapped to the central part of the graph, with counterclockwise rotations innermost (e.g., in red). Rotations of 36° (angle between landmark and maze arm) are mapped to the outer circular arcs (either clockwise or counterclockwise; e.g., in cyan). **G.** Mapping the animal’s 3-D point of regard. Left: three-dimensional schematic of the maze (green), monkey (brown), and point of gaze (red dot). Blue rectangles represent the location of the landmarks. For ease of representation, we define an invisible cylindrical wall running through the landmark centroids. Right: convention for the flattened representation of the point-of-gaze map. When directed further than the distance to the landmark wall, the point of gaze was computed as directed towards this wall; then, in a second step, this wall was flattened as an annulus to create the final 2-D map. **H.** Heat map of the point of gaze, overlaid on a schematic of the maze for one session (monkey S). The regions of interest explored by the animal are the ends of the paths, the landmarks, and the rewarded area.

We trained two rhesus macaques to navigate with a joystick in a virtual 3-D star maze (Fig 1A and 1B, Materials and Methods, and S1 and S2 Movies). The monkeys sought a hidden reward located at the end of one of the five paths, between two landmarks (by convention, the northbound path). For example, on Fig 1B and 1C, the rewarded path is located between the sunflower (northwestern landmark) and the house (northeastern landmark). On each day, new landmarks were used so that the layout was new and unfamiliar. Nothing else in the maze but the landmark layout could be used to infer the reward position because path surface and background were identical across paths. Thus, animals had to start each session without applicable information from past sessions as to the reward's location and learned to find it with respect to new landmarks by trial and error. Each session lasted for 80 trials (± 5 trials). Each trial started at the extremity of a maze path. Animals pushed the joystick to move forward and traveled towards the center (Fig 1C, first panel; a triangle symbolizes the field of view [FOV] of the animal). At the center, animals could rotate the joystick left or right to choose another path to enter (Fig 1C, second panel). The example on Fig 1C shows the monkey entering the rewarded path after a left turn (Fig 1C, third panel). When they reached the end of that path, animals received a juice reward directly in their mouth (Fig 1C, fourth panel). Finally, the animals were reallocated to a different, randomly assigned start (Fig 1C, fifth panel). This latter trajectory did not follow any maze arms, preventing the animals from retracing their steps after a correct choice. Importantly, the landmarks were positioned between the maze arms, thus dissociating the goal from the visual references. In other words, the animals could not directly associate a physical object (landmark) to the reward but had to analyze and memorize the spatial relationships between landmarks and reward. Fig 1D shows the same corresponding five steps as in Fig 1C but from the animal’s perspective (70° horizontal FOV). Overlaid on this view is a representative density heat map of the animal’s point of gaze for 500 ms at each of these steps.

We further computed the animal’s allocentric point of gaze using their virtual self-position and head direction and eye tracking data (Fig 1G). Point of gaze density maps (e.g., Fig 1H, S1 Fig, and S1 Appendix A1, Material and Methods) revealed that gaze was attracted to the rewarded path and the landmarks. During the 500 ms before each action on the joystick (Fig 1D, S1 Fig, S2 Fig, and S1 Appendix A2), gaze anticipated the direction of the subsequent movement (Wilcoxon test, *p* < 0.001 (S1 Fig, S2 Fig, and S1 Appendix A2). Similarly, when being relocated to a new entry, monkeys proactively gazed at the location at which the landmarks would appear (see S2 Fig and S1 Appendix A2) (Wilcoxon test, *p* < 0.001). These patterns of visual exploration are similar to ones described in humans when driving [21].

Animals quickly solved the task. On average, both animals learned to reach the rewarded arm in a dozen trials (S3A and S3B Fig); monkey S performed above chance after 12.3 ± 2 trials (SEM), and monkey K did so after 14.8 ± 2.4 trials. From then to when the upper confidence bound of success reached 90%, less than 10 trials were usually necessary (89% of sessions).

To more closely examine the nature of the animal’s spatial representation, we conducted probe sessions (9 sessions for monkey S and 15 sessions for monkey K). In these sessions, animals first started from only one or two entries (northeastern and southeastern entries) and were only later introduced from the new remaining entries (northwest and southwest). We hypothesized that if animals formed a cognitive map of the maze [22], they would successfully transfer knowledge acquired from the previous entries to the new entries (northwest and southwest). Monkey K was 73% correct after introducing the new entries versus 44% correct at beginning of the sessions; monkey S was 80% versus 55% correct (S3C Fig, Wilcoxon, *p* = 0.01). Thus, after having explored the maze from entries facing eastern landmarks, animals were able to deduce goal location when entering new paths facing western landmarks. Further, performances above the learning criterion were reached significantly faster (within 4 trials for monkey K and within 2.7 trials for monkey S, compared to 14.8 and 12.3 trials; Wilcoxon, *p* = 0.002).

Thus, animals attended to landmarks and used them flexibly depending on their self-position. Their exploratory behavior in a VR setting appeared similar to that described by others in real-world navigation, as it possessed essential properties of wayfinding such as reliance on landmarks and flexible trajectory planning [22].

### What is encoded in hippocampal cell activity?

Of the 270 cells recorded in the full extent of the right hippocampus (128 cells in monkey S, 142 in monkey K; S4 Fig), we focused on 189 cells that fired more than 100 spikes per session (approximately > 0.01 Hz; see S1 Appendix A3, S3 Fig, and S4 Fig). Only successful trials were considered for analysis.

For comparison with rodent studies, we analyzed neural activity as a function of the animal’s current location in the maze (“Position”) and the current virtual head direction (“Direction”). As vision is paramount in primates, we next examined the impact of the current allocentric point of gaze in the virtual maze (Fig 1D). Then, we considered a fourth explanatory variable by constructing a state-space representation of the maze. State spaces are often used in models of animal navigation [23] and may provide a useful framework to account for hippocampal cell activity [19]. Our state space can be thought of as a logical representation of the task as a graph (Fig 1F), with each node being a choice point (stable state of the animal—e.g., “in the center, facing the northeastern landmark”) and each link corresponding to the state change brought about by an action of the animal. Note that in our task, the animal’s actions are discrete: for example, pushing the joystick forward once is enough to make the monkey travel forward from one end (current state *s(t)*) to the other end (state *s(t+1)*) of the current maze arm (similarly, pushing the joystick leftward once is enough to rotate the monkey leftward by 72° if facing a path or by 36° if facing a landmark). Thus defined, this state space describes the resolution of the imposed navigation problem as a series of spatialized action steps. In particular, the state-space graphical representation, by combining the animal’s current view, position, and action, allows distinguishing multiple action contexts for the same position and direction at the maze center.

**Fig 2:**
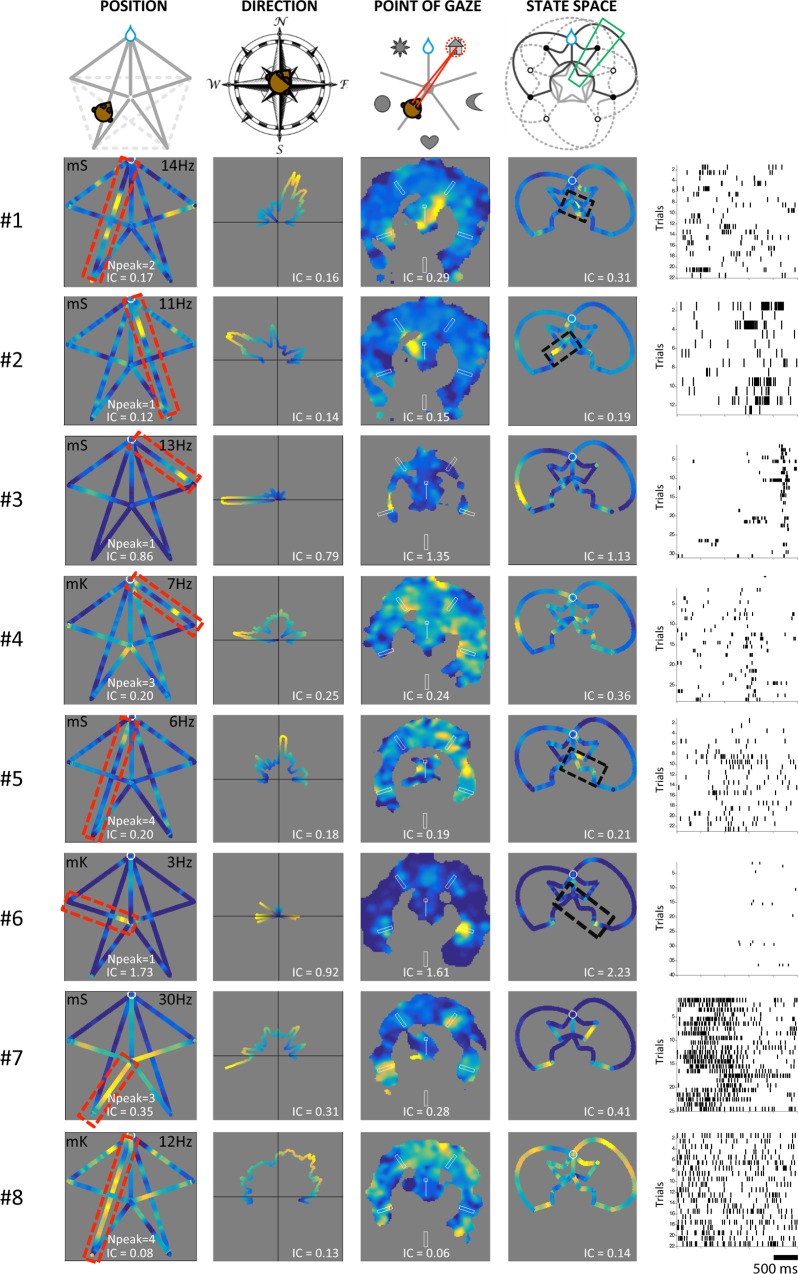
Individual examples of cell activity in the four coding spaces studied. Each space is mapped in a column (columns 1–4). The top row describes the structure of each of the coding spaces: monkey self-position (position), virtual head direction (direction), flattened gaze map (point of gaze), and state space (state space). Note that the state-space graph is drawn so that a sector like the one highlighted in green contains all the moves in which the monkey faces in the same direction (here, towards the northeastern landmark). Rows 2 to 9 represent the activity of eight individual cells that illustrate different firing patterns. The far-right column represents a raster histogram of the activity of each cell for all the laps that occurred in the path highlighted in red on the far-left figure for cells 3, 4, 7, and 8 or in black on the right adjacent figure for cells 1, 2, 5, and 6. In this raster representation, each row corresponds to an individual trial, and each tick represents an action potential, on a time window of 2.5 s. Monkey identity is indicated with mS or mK on the position maps. Underlying data can be found at http://dx.doi.org/10.6080/K0R49NQV.

We chose eight cells to illustrate the diversity of responses across the population (Fig 2). Most cells appear as regular place cells (first column), some with multiple place fields. This might result from the frequent interdependence of direction and position: the strong directional sensitivity of cells 1–5 could account for these fields (second column). However, further examination shows that the strong directional firing could be explained by gazing at specific landmarks (third column). Cells 1 and 2 were recorded simultaneously and exhibited different landmark preferences. Population averages indicate that all landmarks appeared represented by the cells (S6C Fig and S1 Appendix A4). Importantly, this landmark preference was often expressed exclusively along particular segments of the animal’s trajectory. This combination is evident when cells are mapped in the state-space coordinates (fourth column). The graph shows activity on the return paths from reward position to new start as well as activity when the animal is turning in the middle of the maze and passes in front of the landmarks (central rosetta of the state-space graph). This latter activity cannot be visible in the position graph because activity for different directions cancels out when averaged in the center. In this framework, cells 1, 2, 5, and 6 exhibit selective activity for specific segments in the center associated with visible landmarks and/or the animal’s rotations (black boxes in the fourth column). The far right column shows the activity of the cells as raster plots for the corresponding trajectories highlighted either in red or in black. Although most cells showed narrow selectivity and less than three positional fields (S5 Fig), some cells (like 7 and 8) had weak but significant modulation of activity in all four coordinate sets and multiple fields (*n* = 3 and *n* = 4, respectively).

### Sensitivity of the cells to a combination of variables

We evaluated the four coding spaces using standard measures employed in rodents: the information content per spike (IC) and a sparsity index [3,6,24]. The first quantity documents how much spatial information a spike conveys, while the second expresses spatial selectivity. To compare IC across coding spaces, we ensured that the same number of bins was used across cells and spaces and normalized IC with respect to the average IC of 999 surrogate datasets generated by randomly shuffling periods of spiking activity in time (see Materials and Methods). While this method may be conservative [25], it is widely used in rodent literature (e.g., [1–7]) as it effectively conveys information relative to the spatial distribution of the cell’s activity [26].

**Fig 3:**
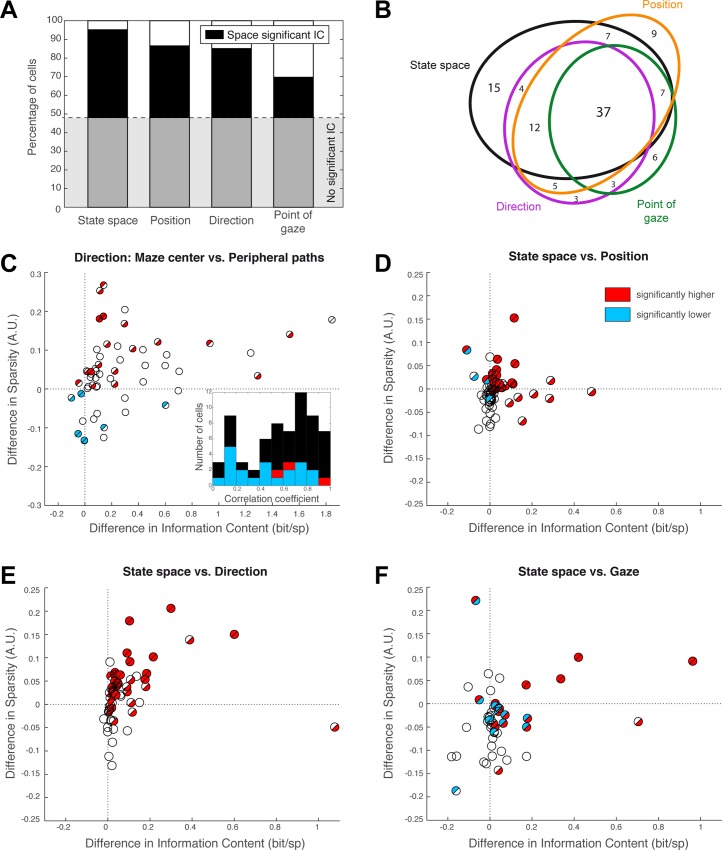
Population statistics. **A.** Proportion of responsive cells (cells with significant information content [IC], above gray line) and proportion of cells significantly coding each of the four spaces (black). Open bars correspond to cells with significant IC in another space. **B.** Representation of the main intersections amongst each subpopulation of cells represented in **A**. Most responsive cells carry significant activity in more than one coding space. **C.** Analysis of the responsive cells’ directional sensitivity and selectivity, comparing activity in the center of the maze to activity on peripheral paths to next start. The difference in information per spike is plotted against the difference in sparsity. Filled half-discs indicate significant differences, as established per cell (top-left half: significant sparsity difference; bottom-right half: significant information difference). Red indicates significantly positive differences (i.e., center > periphery), and blue indicates negative differences (center < periphery). Overall, the activity in the center is both more consistent and more direction specific. Inset: Distribution of the correlations between directional tuning in the center and in the periphery. Significantly high (red) and low (blue) correlations are indicated. **D–F.** Difference in information per spike versus difference in sparsity when comparing cell activity readout in state space to activity readout in position space (**D**), direction (**E**), and point of gaze (**F**). Same graphical conventions as in **C**. Statistical significance was obtained with permutation tests (surrogate spike datasets). Underlying data can be found at http://dx.doi.org/10.6080/K0R49NQV.

Statistical significance was tested by comparing measures derived from actual data with those in the 999 surrogate datasets. Significant IC (*p* < 0.01) was present in at least one of the spaces for 111 out of the 189 cells (59%, responsive cells). The proportion of cells responsive to each space was significantly unequal (chi-squared test, *p* = 0.014). The state space accounted for the largest number (84 cells, Fig 3A), and pitting state space against the other three, there were more cells responsive to state space than to point of gaze (chi-squared test, *p* = 0.012, Bonferroni corrected; the differences between state space and other spaces did not reach statistical significance). Sensitivity to these four variables was not mutually exclusive (Fig 3B).

Note that the simple difference of space dimensionality does not account for this IC difference (S1 Appendix, A5). Population averages of the activity maps (S6A–S6D Fig) revealed that fields across all coding spaces are inhomogeneous, this being likely related to the presence of landmarks (S1 Appendix, A4).

### Direction selectivity

To disentangle direction and position, for each direction-selective cell we compared firing on active center time and peripheral paths to next start. Both IC and sparsity measures were significantly higher in the center, which is the choice point, when compared to return tracks (both *p* < 0.001). Direction tuning was not maintained across positions, since the correlation between direction selectivity in the center and in the return paths was significantly lower in 20 out of 56 cells (inset in Fig 3C, blue bars). This represents 36% of the population and is higher than what is expected by chance (*p* < 0.001, binomial test; note that correlation was only significantly higher in 3 cells, consistent with chance: *p* = 0.16, binomial test). Overall, these analyses confirm on a population level that head direction is rarely if ever coded alone but is combined with variables such as position, point of gaze, and action choices.

### Hippocampal cells encode a fine-grained state-space representation of the maze

How well does a coding space account for cell activity? As a majority of cells were responsive in the state space, we took this space as a reference. Then, for each cell, we compared the difference between the normalized index computed in state space and the corresponding normalized index in another coding space, considering only cells responsive in both spaces. Normalized indexes were obtained by subtracting the average indexes obtained from the surrogate data from the raw indexes in order to prevent any bias due to space structure. The joint distribution of these differences in IC and sparsity is illustrated in Fig 3, where state space is compared to either self-position (Fig 3D), direction (Fig 3E), or point of gaze (Fig 3F). The spike information content for the state space was systematically higher when compared to position, direction, or gaze space (Wilcoxon, all *p* < 0.01). Sparsity was significantly higher (Wilcoxon test) in state space compared to direction space (*p* = 0.006) but not significantly higher compared to position space (*p* = 0.19), although the 15 cells for which the difference was individually significant (as assessed with permutation tests on surrogate data) were sparser in state space (*p* = 0.001). Conversely, sparsity was significantly higher overall in gaze space than in state space (*p* < 0.001), but this effect could not be confirmed when considering only 18 cells for which the difference was individually significant (*p* = 0.68). This pattern of results suggests that cells respond to a combination of variables rather than to one single dimension. This effect cannot be attributed to a modeling bias related to the higher number of dimensions in the state space (S1 Appendix A5 and S7 Fig). As the state space represents every trajectory within each direction in space and a joint action, the results imply that the most informative cells have a fine-grained representation of space that integrates position, direction, and point of gaze into a higher order trajectory representation.

### Cells discriminate different contexts for the same position and direction

**Fig 4:**
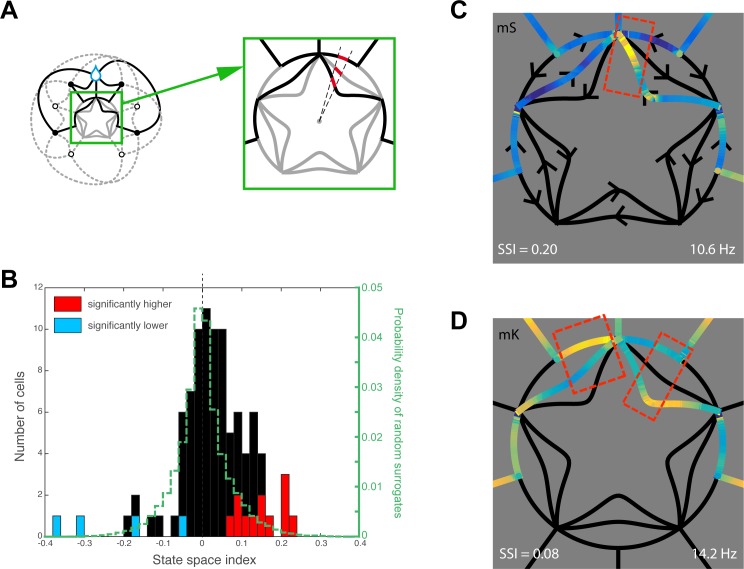
State-space selectivity. **A.** Schematics illustrating the analysis method whereby activities corresponding to the same location (maze center) and direction (dashed sector) were compared on the different state-space transitions (in red). **B**. Histogram of the state-space selectivity indices across the responsive cells. Cells significantly invariant to current transition are in blue; cells significantly context-dependent are in red (permutation tests). The distribution of indices for the surrogate spike sets is shown in dashed green. **C–D**. State-space maps (restricted to the center) of two context-dependent cells. Underlying data can be found at http://dx.doi.org/10.6080/K0R49NQV.

A large part of the state-space graph corresponds to unique combinations of self-position and head direction. To distinguish a joint representation of position and direction from an actual state-space representation that includes trajectory context, we singled out the part of the state space corresponding to the maze center. There, activity corresponds to a single position and direction but differing rotations (Fig 4A), so we could define a state-space selectivity index based on the normalized differences between trajectory moves for the same head direction (see Materials and Methods). Only 4 cells showed significantly low state-space selectivity, as expected by chance (*p* = 0.81, binomial test), but 12 cells out of 111 (11%, *p* = 0.01, binomial test) showed significantly high state-space selectivity, indicating they were also sensitive to the current action of the animal (Fig 4B, red bars). The activity maps of two such context-dependent cells are shown on Fig 4C and 4D, wherein the same joystick move is present at least three times without similar accompanying firing patterns. These cells could encode an abstract representation of the maze, comprising sensory aspects of self-position and direction with respect to landmarks as well as contexts of current (and previous or upcoming) actions.

### Cells encode landmark identity and distance with respect to landmark

**Fig 5:**
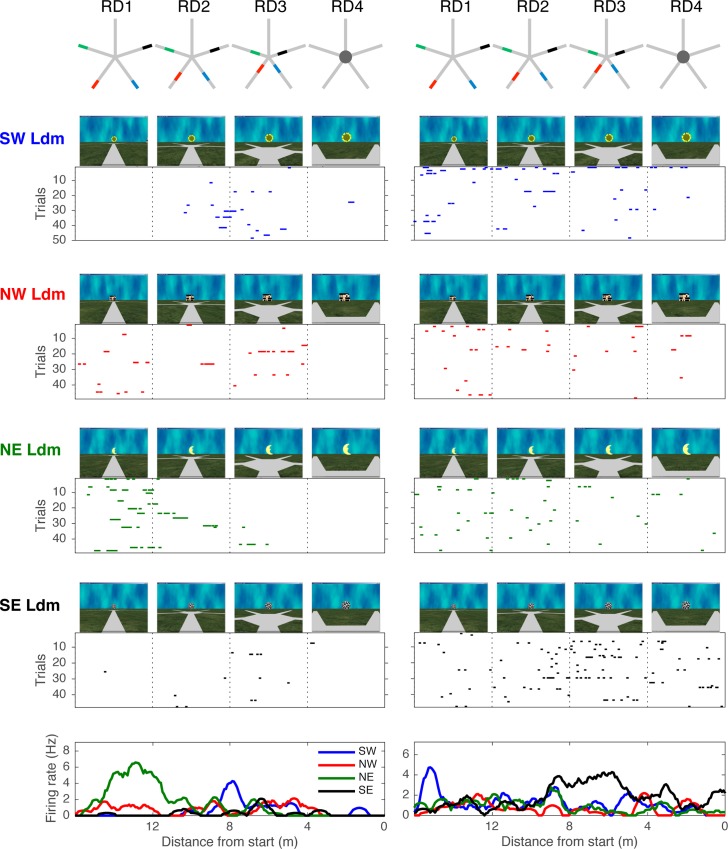
Sensitivity to landmark identity and relative distance. **Left panel**. Activity of a cell for each of the four landmarks viewed at four intervals of relative distances on the entry path (RD1 to RD4, see Materials and Methods). Top row: schema of the maze with these distance intervals illustrated as areas for each landmark (southwestern landmark in blue, northwestern in red, northeastern in green, and southeastern in black). The pictures above the rasters show a still image of the monkey’s view of the landmark at each relative distance symbolized by dotted lines on the raster (12, 8, 4), the last one being at 0. Each raster represents the activity of the cell to each landmark as the animal moves forward in the corresponding path. On these rasters, each line is a trial. The bottom graph shows the average cell activity over all trials. **Right panel**. Activity of a different cell recorded during the same session as the cell shown in **the Left panel**. Underlying data can be found at http://dx.doi.org/10.6080/K0R49NQV.

Landmarks are the only cues available to the animal to get his bearings, and as expected, population averages of the activity maps (S6C Fig) show that gazed-upon landmarks elicit strong responses. Accordingly, Fig 2 shows that some cells (cells 1, 2, 3, and 6) exhibit increased activity when the animal was looking at a specific landmark. However, the cells also seemed to exhibit a modulation of their activity from different positions with respect to the viewed landmark. To quantify systematically whether cells exhibited landmark preference and how this interacted with other variables such as distance from landmark, we compared the activity to the four different landmarks in four intervals of relative distance with respect to the landmark (4 x 4 factorial design, see Materials and Methods). In this analysis, we excluded the activity on the peripheral paths as landmark position, and aspect was not stable in the scene. Thus, we selected only the portion of trajectories on the start paths for which the relative distances were identical across each landmark. A two-way ANOVA revealed a main effect of landmark identity (30 cells out of 111 responsive cells) and relative distance (64 cells out of 111 responsive cells). The main effect of landmark identity was confirmed by a one-way ANOVA on the activity to the four landmark views at the center only (12 cells out of 111 responsive cells, which is a higher proportion than expected by chance; *p* = 0.01, binomial test). While the two-way ANOVA emphasizes the coding of relative distance by the cells, it also confirmed that many cells conveyed a combination of information between the identity of the landmark viewed and the position as previously unraveled by the state space. Twenty-nine cells coded a combination of these two factors with a significant interaction (14 cells) or both factors significant (15 cells). Both proportions are higher than chance (*p* < 0.001, binomial test). Fig 5 shows the activity of two cells recorded concurrently during the same behavioral session. The cell on the left shows a high activity for the northwestern landmark viewed from distance RD1 (F(9,756) = 4.4076, *p* < 0.001); the cell on the right shows a high activity for the northeastern landmark viewed from distance RD3 (F(9,756) = 2.1243, *p* = 0.025). In sum, the results indicate that cells are sensitive to the identity of the landmark viewed by the animal while also being modulated by the distance from the animal to the landmark. Thus, this analysis comports with the conclusions of the state-space analyses.

### Landmark-triggered activity and viewpoint dependence

Some cells (e.g., cells 1, 2, and 5 in Fig 2) seem to respond similarly to one landmark from different viewpoints (trajectories highlighted in the black and red boxes). Nevertheless, the way cells relate to landmarks is usually more complex than a sensory response, as expected from the foregoing analysis. For example, in Fig 2, five of eight cells (cells 3 to 7) show activity to a landmark from only one or two viewpoints amongst several possibilities.

**Fig 6:**
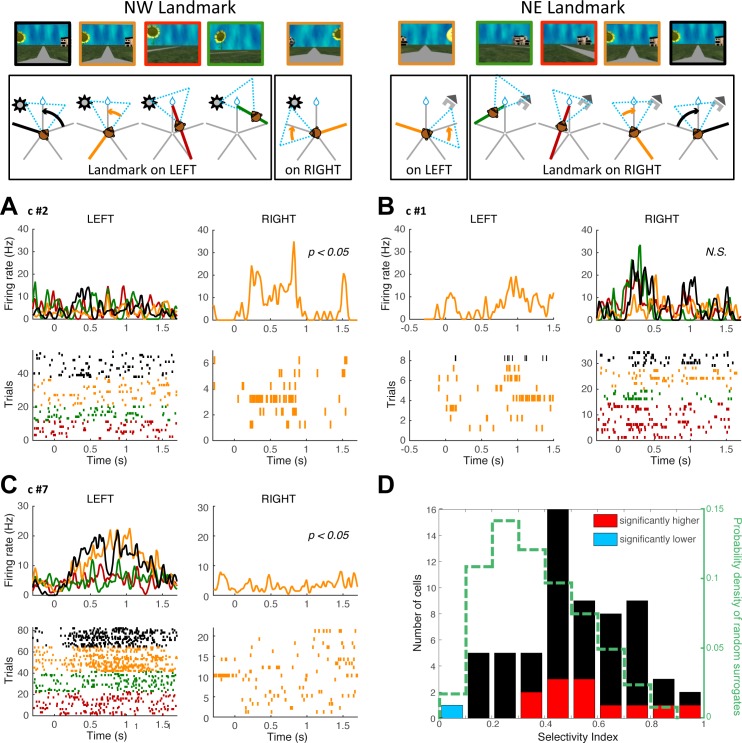
Landmark viewpoint-invariant versus viewpoint-dependent cells. Top row: schematics of the monkey’s five different viewpoints for either the landmark immediately left or the landmark immediately right from the reward location. For every path, the landmark appears either on the animal’s left or right. **A–C**. Individual examples of cell activity (average and trial-by-trial raster histogram; cells numbered as in Fig 2) aligned on the landmark’s left or right entries in the animal’s field of view. The color codes correspond to the activity on the individual paths identified in the top row. Cells displayed in **A** and **C** discriminate between different viewpoints, while the cell displayed in **B** does not. **D.** Path selectivity index calculated for the different viewpoints of the landmark left or right of the reward (best landmark for each cell). In red are cells for which the index was significantly higher from chance (viewpoint dependent), and in blue are cells for which the index was significantly lower than chance (viewpoint invariant). The distribution of indices for the surrogate spike sets is shown in dashed green. Underlying data can be found at http://dx.doi.org/10.6080/K0R49NQV.

To investigate this viewpoint dependence at the population level, we compared activity collected for different trajectories exposing the same landmark. This was reliably possible for the two landmarks neighboring the reward that were visible in five paths (Fig 6, top row schematics). As such, we analyzed 42 cells exhibiting a significant activity to the appearance of the northwestern or northeastern landmark and significant IC for the point of gaze. Fig 6A–6C shows the activity of three cells of Fig 2 as a function of the path. To test whether the cells’ immediate response to a landmark varied with viewpoint, we computed for each landmark a path selectivity index (see Materials and Methods). The indices were computed on a 500 ms epoch beginning with the appearance of a landmark in the visual scene, with a 120 ms offset to account for visual latency. We found that only one landmark-responsive cell was significantly path invariant (Fig 6B, corresponding to cell 1 in Fig 2, 5% of the 42 cells, *p* = 0.37, binomial test), whereas 12/42 cells (29%, *p* < 0.001, binomial test) discriminated significantly amongst different viewpoints of the same landmark (Fig 6D, single examples in Fig 6A and 6C). The distributions of the actual versus surrogate selectivity indices were also significantly different (*p* < 0.001, Kolmogorov–Smirnov test), confirming that at the population level, viewpoint selectivity in our data is far more represented than viewpoint invariance. This viewpoint dependence would support egocentric updates of self-position in space.

### Visual exploration and hippocampal activity dynamics

**Fig 7:**
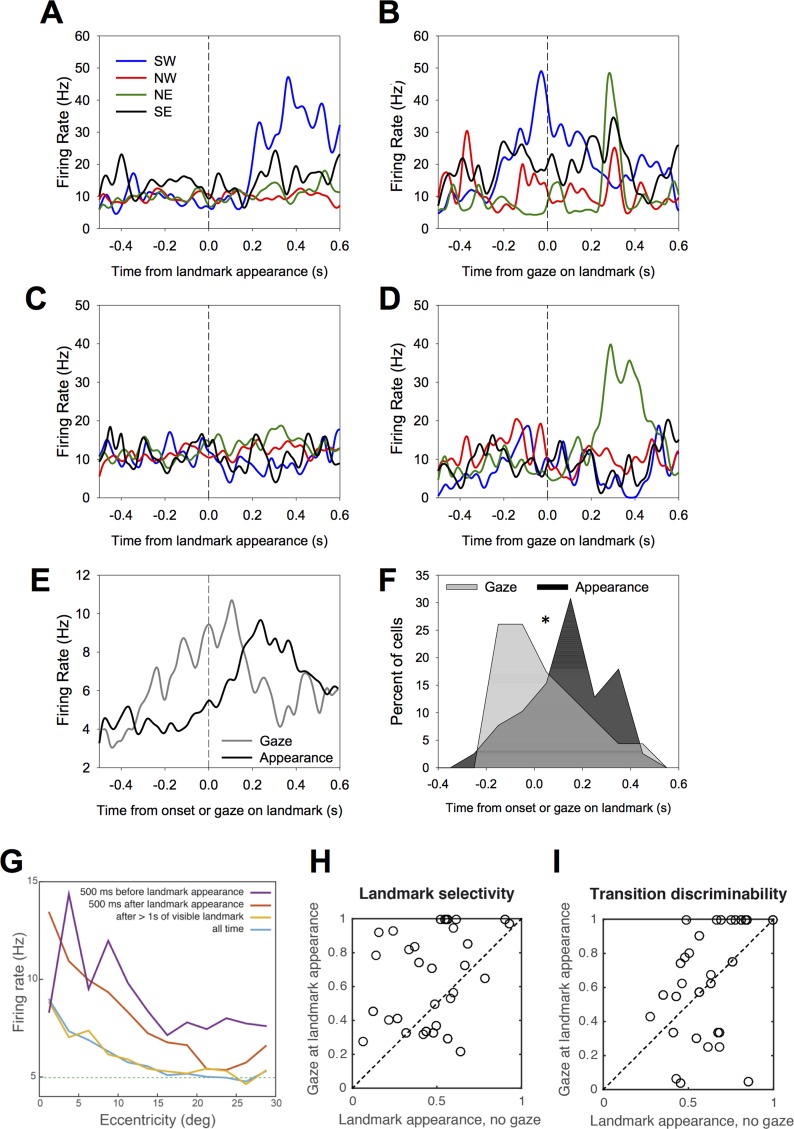
Modulation of cell selectivity by the point of gaze. **A.** Mean activity of an example cell towards each of the four landmarks aligned on the entry of the landmark in the field of view or **B** aligned on the animal’s saccade towards the landmark. This cell has a preference for the southwestern landmark, and its activity peaks at the time of the saccade on the landmark. **C–D.** A second example cell with the same conventions as in **A–B**. This cell shows a higher activity to the southeastern landmark when the animal gazes at it (**D**) compared to when it enters the visual field (**C**). Those two examples illustrate two patterns of activity: (1) the activity of the cells peaks around the moment that the eyes reach the landmark, and (2) the gaze increases the firing rates associated with one landmark. **E.** The temporal dynamics of the mean cell activity with respect to landmark appearance or gaze on the landmark. **F.** Distribution of the latencies with respect to landmark appearance or foveation. **G.** Mean cell activity as a function of landmark eccentricity on the retina, considering four set of epochs relative to landmark appearance (see main text). Firing was always modulated by foveation but more so when the landmark recently appeared (red). Cells fired in anticipation of the landmark (purple), but activity was higher if the monkey had previously made a saccade close to its expected point of appearance. Dashed line: average firing rate. Note that the vertical scale does not begin at zero. **H.** Landmark selectivity indices calculated for activity aligned on landmark appearance when gaze was not directed to them versus activity aligned on landmark foveation following its appearance. **I.** Same as **H** for the path selectivity index. Underlying data can be found at http://dx.doi.org/10.6080/K0R49NQV.

How is the neural response to the landmarks related to the animal’s visual exploration of the scene? Some cells appear triggered by the first entry of a landmark in the animal’s FOV (Fig 2, cells 1–5). This interpretation is not possible for cells 6 or 7 because the firing rate increases in a start arm, well after the landmark has entered the FOV during the preceding return move. To clearly assess the relationship between the gaze on landmarks and cell activity, we aligned the activity of each cell to its best-driving landmark, either on its appearance in the FOV (landmark “onset”) or on its first foveation (Fig 7A–7D, see Materials and Methods). Then, we computed the average response for both alignments (Fig 7E). Gaze-aligned activity significantly rose until the saccade to the landmark and peaked shortly after. The distribution of the latencies of the responses to the best landmark are left skewed when aligned on gaze and right skewed when aligned on landmark onset (Fig 7F, see Materials and Methods). For a small but nonnegligible proportion of cells, the response latency even preceded the landmark’s entry in the FOV, suggesting a predictive representation of its location in space prior to becoming visible. To further examine the nature of this anticipatory activity, we analyzed the cell activity with respect to the eccentricity (distance in degrees to the fovea) of the best-driving landmark. We computed the population average, considering four different subsamples of the data: (1) all the 500 ms epochs that followed landmark appearance in the visual scene, (2) all the 500 ms epochs that preceded landmark appearance, (3) periods starting when the landmark had been visible for at least 1,000 ms, or (4) the whole dataset. For landmarks still outside the field of view (second subsample), landmark eccentricity is computed as it would appear if the field of view was complete (180° x 180°). Overall, cells showed a modulation of their activity by landmark eccentricity (Fig 7G, blue line). This modulation was increased by the recent appearance of the landmark (red line). Nevertheless, and in accordance with Fig 7E and 7F, cells could fire for a landmark still invisible, even if it were not to appear close to the fovea (purple line; activity was still higher if the monkey has previously saccaded close to the expected point of landmark appearance). This observation suggests that the animal could anticipate landmark appearance. Above all, it appears that the concept of receptive field does not apply to these cells, which is expected if they signal a context-sensitive, higher-order conjunction related to the completion of the task.

To evaluate the added value of the gaze with respect to landmark onset alone, we compared (a) the firing rate to each landmark during the 500 ms period following its appearance (excluding instances when a saccade was made at the landmark during that period) and (b) the firing rate within the 500 ms after landmark foveation. On this basis, we computed the path selectivity index (as for analysis in Fig 6) and a landmark selectivity index, which evaluates how much a cell discriminates between the different landmarks. Landmark selectivity was significantly enhanced if the landmark was foveated compared to appearing in the visual periphery (one-sided logit-Wilcoxon, *p* < 0.001, Fig 7G). Thus, a directed gaze correlated with a greater extraction of information from the landmarks. In contrast, path selectivity was only moderately improved by direct gaze (Fig 7H, *p* = 0.036), consistent with a ceiling effect whereby once the maze layout is learned, path identification would not need systematic visual checks at visible landmarks. Overall, these observations show that the firing patterns during ocular exploration are not simply triggered by low-level characteristics of the optic flow but reveal an active search for spatial information.

## Discussion

For the first time in the monkey we jointly examined navigation behavior, gaze fixations, and hippocampal activity to detail a comprehensive picture of the primate coding of space during a goal-oriented navigation task. We found that animals in our settings formed a representation similar to a cognitive map of the virtual maze in that they computed a trajectory from a new starting point during probe trials. The analysis of eye movement revealed that animals explored the VR environment in congruence with upcoming actions, suggesting a reliance on acquiring visual information to guide their moves.

Very importantly, this ability to compute a trajectory via awareness of self-position, direction, and action contexts was mirrored by hippocampal cells’ activity, best understood in a fine-grained state space. Indeed, many of the cells reliably responded as the animal viewed or gazed at a specific landmark but only on selective segments of the trajectory bearing a view to this landmark. Thus, rather than behaving as simple place cells or target-of-gaze cells, hippocampal neurons combined different aspects of the animal’s current sensory state with the goal-related action context. Crucially, some “state-space selective” cells discriminated between two identical position-orientations (thus, two identical sensory inputs) and even two identical prospective actions when they were part of different action contexts. This implies that cells did not code for the visual properties of the landmark or for a systematic association between a cue and an action but rather expressed self-position in an abstract, multidimensional representation of the maze.

### Wayfinding in virtual reality

Our findings were obtained in VR, and this technique has recently been a welcome substitution for the real world when exploring the neural basis of spatial cognition in humans and in animals [3–8,27–32], as it allows a fine control of the environment. Nevertheless, this technique raises interpretative issues as to how well navigation in VR reflects its real-world counterpart. In a virtual setup like ours or those used for human fMRI, subjects lack real inputs from motor, vestibular, and proprioceptive systems. Importantly, the optic flow activates a common neural circuitry with vestibular input that underlies self-motion encoding [33]. Our virtual setup, which primarily simulated extrafoveal territory (70° FOV), shares common properties with stimuli that are known to produce vection-like phenomena [34]. It is thus likely to produce an illusion of self-motion. The animals’ anticipatory gaze behavior, similar to human drivers [21,35], confirms this interpretation. Previous experiments using VR in monkeys had animals travel well-learned routes [27], execute repetitive motion sequences [28], or search explicit visual goals [36]. Our animals’ use of landmarks—inferring the position of a goal dissociated from the landmarks themselves as in triangulation, a hallmark of hippocampal function [22,37]—is novel, and the first replication in the monkey of aptitudes already shown in rats [31] and humans [30,32] in a VR world.

### Nature of the hippocampal signals: Space in context

The signature of hippocampal activity in rodents is the coding by place cells of the animal’s current position [1]. Previous results in primates, including humans, showed that hippocampal cells encoded spatial views [10,11], place [9,14–16,28,38], or a mixture of place and view [8]. In a controlled VR wayfinding setting, the present findings show that hippocampal cells display a fine-grained tuning that preferentially codes one of the landmarks being viewed by the animal and its current self-position relative to the landmark. This shows that primate hippocampal cells also carry position information as rodent place cells do. Beyond that, however, we found that this tuning included trajectory-related contextual aspects and was best captured in a state space because the cells showed higher spike information content when compared to other coding spaces such as self-position, head direction, or point of gaze. Crucially, analysis of the firing rate in the center of the maze showed that the context truly gave additional information compared to a simple combination of position and direction (Fig 4). Further, as shown in Fig 5 and Fig 6, we found more cells than expected by chance that coded a combination between landmark viewed and distance to the landmark or that discriminated trajectories bearing views of the same landmark. Lastly, we observed that gazing on landmarks increases the cells’ selectivity to the path or trajectory bearing the landmark (Fig 7). In combination, these results imply that hippocampal spatial memory involves more than a simple spatial relation to the environment but rather a sensorimotor trajectory scheme in a goal-reaching context. Hippocampal cells responding to the spatial views have been previously reported [10,13]. The identity of the views represented in those cells had precedence over position in explaining their activity. While our cells bear a resemblance to the ones described by Rolls and colleagues [10,13], we found that complementary information relevant to position and action was also robustly encoded in the cellsʼ firing. This difference might be due to the task we used, in which the current viewpoint guides the next actions of the animal. Such imperative contingencies were absent in the studies by Rolls and colleagues. Our findings echo findings in rodents in which the activity of place cells depends on the goal of the trajectory [39,40] or its retrospective and prospective components [41]. In addition, our results imply that hippocampal cells in the primate represent more than the spatial view [10,11] or its associated reward value [42]. How can we interpret our results with respect to the animal’s behavior? As the same viewpoint for the same landmark, same heading, and same prospective action can be reliably discriminated for different trajectories, cells do not code the sensorimotor properties of the task only. Rather, we hypothesize that cells embody a dynamic knowledge about the self-position with respect to the landmarks in a contextual fashion, depending on the current trajectory.

In fact, this conclusion extends previous reports that the rat hippocampus shows trajectory-dependent firing [41,43,44]. To what extent these effects are due to interactions within a broader network such as the prefronto–thalamo–hippocampal circuit [44] remains to be examined in the monkey.

### The importance of gazing at the landmark

Our findings also underscore the power of the relationship between visual exploration and hippocampal activity. Indeed, visual exploration of the environment led by saccades and fixations is part of the primate-specific repertoire of active sensing and is supported by dedicated visual processing areas shared by human and other primates [45,46]. In VR studies like ours, directions can be primarily obtained by visual information only, and accordingly, we observed that the point of gaze importantly modulates the hippocampal activity, as already shown by Rolls et al. [11,13]. This is seen both in single examples (Fig 2) and by the high number of cells with spike information modulated by the point of gaze (Fig 3).

The temporal dynamics of cell activity with respect to the landmark-directed saccades reveals that firing mostly follows landmark appearance but precedes the eyes reaching the landmark, suggesting an anticipatory identification of the landmark (Fig 7E–7G). Although the selectivity of hippocampal cells to the identity of the fixated landmark is reminiscent of object fixation cells in the inferotemporal cortex [47–49], hippocampal cells discriminate positions from which the landmarks are gazed at (Figs 4, 5 and 6). Further, we showed the firing rate at fixation was greatly modulated by the time period during which the landmark was fixated, with activity decreasing as the landmark was fixated long after it was already visible. This pattern is coherent with a task-contextual modulation of the cells. Moreover, landmark and path selectivity tuning is enhanced through foveation, suggesting that gaze information enhances coding of self-position. In sum, our data suggest that the counterpart of place cells in primates, as compared to rodents, is expressed as activity related to point of gaze in conjunction with other variables essential to navigation, such as position and identity of visual elements at key instants of trajectory planning.

### Differences and similarities across mammalian hippocampal codes

Recent rodent studies in VR provide a useful common framework to situate our findings and make cross-species comparisons [3–5]. Approximately 50% of our cells showed spatial selectivity like that in rats [3–5] and similar to that obtained in real-world settings [3,50,51]. Our results further confirm that spatial coding can be obtained in absence of vestibular and proprioceptive input. In addition, the number of fields per cell (average = 2.7) was greater than the number of fields described in the rat (about 1.5), but our maze differs from the single alleys and square or round open fields in the foregoing studies. Studies conducted in real, complex environments bearing path repetitions observe many neurons bearing more than one place field [52–54]. The nature of our task environment may thus have played a role in the multiplicity of hippocampal fields.

In rodents, direction selectivity was thought to be exclusive to one-dimensional mazes; however, it was recently demonstrated to be present in two-dimensional environments [3,7]. Our setup did not allow us to characterize direction selectivity in a strict independent way because direction and position often covaried. Thus, our results on the matter have to be taken with a note of caution. However, firing in the center of the maze displayed direction-dependent activity. Taken together, these findings contradict earlier results showing consistently direction-independent responses in 2-D environments [55,56] and further demonstrate that direction selectivity is an essential property of place cells—as recently shown in VR in the rat [3,7] and in the real world in bats [57]—and can be independent from vestibular cues. Note that direction selectivity was not preserved across spatial positions, suggesting its sensitivity to other variables such as visual cues or actions. Hence, we also observed that selectivity is stronger at choice points than at other places.

Lastly, the strength of our cells’ signals (amplitude of firing rate, number of cells recruited) is more on par with that in the rodent or bats than in previous VR monkey studies, which appeared to engage hippocampal activity rather poorly [28,38]. Our animals had to learn anew the significance of the landmark configuration during each recording session, whereas the aforementioned studies relied on a shuttle behavior between fixed reward zones, suggesting that dissimilar learning requirements account for the different firing rates.

Studies in humans of hippocampal neuronal activity during spatial navigation are rare. Two studies [8,9] provide arguments for homologies in hippocampal processing between nonhuman to human primates. In particular, Ekstrom and colleagues [8] described many cells that showed an interaction between place, goal, and view. The state space–selective cells observed in our study bear resemblances to the conjunctive cells they reported. Such a relationship could account for the interaction of place and goal found in the human hippocampus [8]. This conjunctive type of coding appears ubiquitous across mammalians, as our findings are consistent with the encoding of task-related demands by hippocampal neurons in rodents [58].

In sum, we show that space representation in primates embodies self-position with respect to the target of gaze and further carries cognitive information with respect to the current trajectory to a goal. These results clarify ambiguous results previously obtained in primates that suggested hippocampal neurons did not convey self-position but instead a spatial view [10,11] or conveyed self-position but no cognitive information [12]. They also bridge the gap between results in rats and humans by showing that place cells support self-position in a cognitive map in primates as well and clarify how this coding is constructed through visual exploration and self-movement.

For long the dominant view of hippocampal function has been influenced by the concept of place selectivity, with the hippocampus viewed as a “neural GPS,” highlighting the actual position of the animal on an internal map. In contrast, our results, clarifying and considerably extending the early advances of Rolls and colleagues, suggest a different view of the hippocampus wherein distinct elements of sensory, motor, and cognitive information are linked in order to build a memory (here, a space-related one). In this framework, place fields appear as a mere projection of this memory onto physical space. These results support a new view of hippocampal function and are of relevance for the understanding of the organization of human hippocampal function and memory.

## Materials and methods

### Ethics statement

Our study involved two nonhuman primates. All experimental procedures were approved by the animal care committee (Department of Veterinary Services, Health & Protection of Animals, permit no. 69 029 0401) and the Biology Department of the University Claude Bernard Lyon 1, in conformity with the European Community standards for the care and use of laboratory animals (European Community Council Directive No. 86–609). Further, our procedures were examined by CELYNE, the local ethics board, which approved the in vivo methods used in the laboratory. We minimized animal suffering and maintained their well-being by using anesthetics and pain management during surgeries for recording chamber implantation. During the experiments, animal’s behavior and well-being was monitored. No animal was euthanized after the experiments. Rather, both animals had their implants removed under general anesthesia. One of the monkeys was placed in a sanctuary for monkeys (Natuur Hulp Centrum, Belgium), while the second animal will be placed as soon as possible.

### Behavioral methods and setup

Animals were head restrained and placed in front of a large screen (152 x 114 cm) at a distance of 101 cm. They were further equipped with active shutter glasses (Nuvision) coupled to the computer for 3-D projection (DepthQ projector, Infocus) of a virtual world (Monkey3D, Holodia, S1–S3 Movies). The projection parameters were calibrated to render objects’ size real by calibrating disparity using the actual interpupillary distance of the monkeys (3.1 cm for monkey K and 3.0 cm for monkey S). We confirmed the animals perceived images with the depth of stereoscopic projection by measuring vergence as a small object moved from an apparent 50 cm in front of the screen to 150 cm behind the screen. To this end, two small infrared cameras were mounted above each eye and the movement of the pupils of each eye was monitored (ASL). The cameras further allowed monitoring the animal’s gaze through the task. Animals learned to navigate via the joystick towards a reward hidden at the end of one of the star maze’s arms (Fig 1B, S1–S3 Movies). The star maze had a radius of 16 m and speed of displacement was 5 m per second. This velocity was chosen to optimize the number of rewarded trials in a session and prevent the animals from getting too impatient. During a shaping period that lasted 6 mo, animals learned to find the reward targets whilst operating a joystick that controlled a sphere on the screen. Once they had mastered this task, they were introduced to a 3-D version of this task. Then, they were introduced to a simple Y maze in which they had to move the joystick to approach the sphere. Next, landmarks were introduced along the Y maze, and animals were trained with the sphere in presence of the landmarks. Then, the sphere was removed and animals were rewarded when they went toward the end of the arm where the sphere was last. To this end, they had to use the landmarks. At this point, they were introduced to the full star maze. For one animal that would not go to the end of an arm if a sphere was not there, a different strategy was adopted. We replicated the sphere five times and changed the rules such that there was a sphere at each end, but the animal had to find “the one” which would give a reward and blink when approached. Once this step was learned, the spheres were removed for him as well. Finally, animals were trained to learn new landmark arrangements every day. We used a star-shaped environment rather than using an open field to ensure multiple passes through the same trajectories and to avoid locations with too sparse data. Each day, the animals had to locate a new position of the reward with respect to new landmarks. Each trial began with the animal facing the maze from one arm end. The joystick allowed the animal to move to the center and turn left or right to choose and enter one arm. Once the animal reached the end of the arm, it was given a liquid reward only if correct and then brought to a randomly chosen new start whether the trial was correct or incorrect. Fig 1 presents the sequence of a trial from above (1C) and from the animal’s perspective (1D).

### Mapping the animal’s point of gaze in the allocentric reference frame

We computed the point of gaze in an allocentric frame wherein objects (landmarks) or positions in space towards which the monkey gazed were mapped relative to a top view of the maze (Fig 1G and 1H). To calculate the point of gaze, we combined the X and Z eye-tracking data with the X and Y virtual position of the animal in the maze and the orientation of the camera viewpoint. The points of regard above the horizon were mapped onto a vertical circular wall enclosing the landmarks; this wall was then flattened into an annulus in the map. This map represents where the animal is gazing in the spatial scene, not the craniocentric eye position. The coordinates obtained were then used to compute the firing rate of the cells as a function of the animal’s point of regard (gaze spike map density, Fig 2, third column).

### Electrophysiological recordings

For a period of approximately 6 mo, each animal underwent daily recording sessions during which electrodes were lowered to the target areas (see S4 Fig). Recordings began if individual cells were present on the contact electrodes, and the task was then started. Individual cells were pre-sorted online and re-sorted offline (offline sorter, Plexon Inc.), and only cells whose waveforms possessed reliable signal-to-noise ratios (two-thirds of noise) and whose activity was stable in time such as illustrated in the rasters in Fig 2 (far right panels) were included in the database. The recording sites were located in CA3 (*n* = 99), CA1 (*n* = 101), or the dentate gyrus (*n* = 8). See S1 Appendix A3 for a detailed description of the recording methods and S4 Fig for anatomy.

### Data analysis

All data were analyzed with custom Matlab scripts.

#### Activity maps

We computed each cell’s mean firing rate for each spatial bin, simply dividing the number of spikes recorded in that bin by the total time spent in it. Only bins comprising at least four successful trials were kept. For display (Figs 2 and 4), a smoothing procedure was applied: the instantaneous firing activity was slightly smoothed with a Gaussian kernel of SD = 100 ms before computing the map. When comparing spaces, no smoothing was used and bin sizes were adjusted so that each map contained a similar number of bins (~200).

#### Permutation statistics

To test the statistical significance of any index computed on the spike data, we created 999 surrogate data sets in which we divided the recording time into chunks of 5 s that we randomly shifted. This procedure decorrelated the spikes from the animal’s behavior while essentially preserving the structure of spike trains (e.g. spike bursts). All analyses were run on actual and surrogate data, and for any tested variable, the rank of its actual value among the set of 1,000 (actual + 999 surrogate ones) was used to extract a statistical *p*-value (bilateral test).

#### Information Content (IC)

For each individual cell, we iteratively adjusted the spatial resolution of each map to get as close to 200 valid bins as possible for each coding space (position, direction, point of gaze, and state space). Bins were considered valid if they included more than 400 ms of time in successful trials. We computed the information content in bits per spike with the following formula [24]:

$$I=\sum_{i}\frac{\lambda_{i}}{\bar{\lambda }}\;\log_{2}\;\left(\frac{\lambda_{i}}{\bar{\lambda }}\right)\;p_{i}$$

where *λ*_*i*_ is the firing rate in the spatial bin *i*, $\bar{\lambda }$ is the mean firing rate, and *p*_*i*_ is the fraction of the time spent by the animal in bin *i*. IC is zero for a homogeneous firing over the *M* bins; it is equal to log_2_(*M*) when a single bin contains all the spikes and the animal spends an equal amount of time visiting each bin. To avoid potential bias, we normalized the IC by subtracting from it the mean IC of the 999 surrogate datasets (see above paragraph).

#### Sparsity index

Following standard procedures [3,59], we estimated sparsity by the ratio of L1 norm over L2 norm and defined as sparsity index

$$s=\left(M-\frac{{\left(\sum_{i=1}^{M}\lambda_{i}\right)}^{2}}{\sum_{i=1}^{M}\lambda_{i}^{2}}\right)/(M-1)$$

where *M* is the number of spatial bins and *λ*_*i*_ the firing rate in bin *i* as above. The sparsity index *s* is 0 for a homogeneous firing map and 1 when a single bin contains all the spikes.

#### Directional correlations

For each cell, we correlated the directional firing curves using the uncentered index:

$$\rho =\frac{\sum_{i\in I}{\lambda_{i}\tilde{\lambda }}_{i}}{\sqrt{\left(\sum_{i\in I}\lambda_{i}^{2})(\sum_{i\in I}{\tilde{\lambda }}_{i}^{2}\right)}}$$

*λ*_*i*_ being the firing rate in the center, ${\tilde{\lambda }}_{i}$ the firing rate in the return paths, and I the set of directional bins commonly valid for the aggregated time spent in the maze center and its counterpart spent in return paths.

#### ANOVA for landmark identity and relative distance

Each path facing a landmark was parsed into four identical segments, and firing rates were collected for each of the animal’s laps on each of the four entry paths. Thus, we constructed a 4 x 4 factorial layout in which activity to each of the four landmarks was compared at four symmetrical relative positions with respect to the center of the maze. Relative distances for each landmark were defined as the same distance and frontal angle of view with respect to the landmark: relative distance RD1 corresponds to activity between 16 and 12 m from the center, relative distance RD2 corresponds to activity between 12 and 8 m from the center, relative distance RD3 corresponds to activity between 8 and 4 m from the center, and relative distance RD4 corresponds to activity between 4 and 0 m from the center.

#### Path selectivity index

For each landmark neighboring the reward (northwest and northeast), we identified the five trajectories for which the landmark appeared in the FOV and computed the firing rate from –400 ms before to 500 ms after that onset with a 10 ms resolution. Then, we selected 42 cells which displayed a significant activity to either the northeastern or northwestern landmark by the following criterion: the activity evoked by the landmark had to exceed the mean baseline activity plus 2.5 times the S.D. calculated on the 400 ms baseline preceding the landmark onset. If that criterion was satisfied, we computed a path selectivity index using the mean firing rate during the 500 ms for each path on which the landmark appeared with the following formula:

$$S=\left(N–\frac{\sum_{k=1}^{N}\nu_{k}}{\underset{k}{\max}\;\nu_{k}}\right)/(N-1)$$

where *N* is the number of trajectories (here, *N* = 5) and *ν*_*k*_ is the mean firing rate for trajectory *k*. The selectivity index *S* is 1 when only one trajectory elicits a response, and 0 when all trajectories elicit the same response. For each cell, we considered the value corresponding to the most informative landmark. We compared the distribution of the indices for the actual data compared to the one computed with shuffled spike data.

#### Landmark selectivity

We computed a landmark selectivity index using the mean firing rate for the 500 ms activity after landmark onset, with a 150 ms offset accounting for the latency of the response for each of the four landmarks (northwest, northeast, southwest, and southeast—the landmark opposite to the reward should not be visible in correct trajectories). We used the same method and formula as above (path selectivity) to calculate the index and its selectivity, here with *N* = 4 landmarks.

#### State-space selectivity

Considering only periods when the animal was in the center of the maze, we computed for each 3.6°-wide angular bin *i* the firing rates $\lambda_{i}^{a}$ across the three possible rotational moves (*a* = 1,…, 3). We required that each bin comprised at least 400 ms of time spent in each rotation of at least two different types. We defined the state space selectivity index as:

$$SSI=\frac{\sum_{i}\;\left(\underset{a}{\max}\;\lambda_{i}^{a}-\underset{a}{\min}\;\lambda_{i}^{a}\right)}{\sum_{i}\;\left(\underset{a}{\max}\;\lambda_{i}^{a}+\underset{a}{\min}\;\lambda_{i}^{a}\right)}$$

summing only over well-defined bins, then centered this index by subtracting the mean of the indices obtained on the surrogate spike sets.

#### Landmark foveation

When gaze was detected inside a ±10° zone in azimuth and elevation centered on each landmark for more than 100 ms, we classified the landmark as foveated. Epochs of foveation usually began with saccades directed at the landmark but sometimes corresponded to the landmark entering a portion of space the animal was already looking at (anticipation of the landmark).

#### Latencies of responses to the “best” landmark

For each cell, we identified the landmark for which the activity of the cell was the maximal. Then, we calculated the latency of the cell’s response by identifying the moment following the appearance of the landmark for which the cell responded significantly above baseline with the following criteria: the firing rate had to be higher than the mean firing rate during the baseline (–500 ms to –150 ms preceding stimulus onset), plus 2.5 SD, for a period of 100 consecutive ms. We calculated the latencies for activity aligned on landmark appearance or for the activity aligned on landmark foveation.

### Supporting data

See S2 Appendix for a description of the spike and behavior data files hosted on CRCNS (http://dx.doi.org/10.6080/K0R49NQV) as well as some Matlab scripts necessary to work with them.

## Supporting information

S1 Fig Point of gaze in the allocentric maze space. Point of gaze in an allocentric frame representing the maze from the top. The five rectangles represent the five landmarks, with the one highlighted in white, being the landmark (ldm) that appears on the left or on the right of the animal. The inset represents the actual position of the monkey in the paths within the maze (star arms, and passive returns from the north arm end to each of the other 4 arm ends), for short passive return, long passive return, and center rotation.
(TIF)

S2 Fig Horizontal eye position before a turning action. **A**. Distribution of the mean horizontal eye positions for the 300 ms preceding a turn to the right (in blue) or the left(in r ed) for monkey K (left) and monkey S (right). **B.** Mean horizontal eye position for the 300 ms preceding the second turn of a series of two turns to the right (in blue) or the left (in red) for monkey K (left) and monkey S (right). C. Mean horizontal eye position during the passive return journey towards the right or the left for monkey K (left) and monkey S (right).
(TIFF)

S3 Fig Learning curves: performance as a function of trials. **A.** Average probability of a correct response over the course of a testing block of 80 trials. **B.** Learning curves in three representative individual sessions in monkey K (top) and monkey S (bottom). Dots illustrate trial outcome (blue: rewarded, red: non-rewarded). The solid blue line represents the probability of a correct response, and the dotted lines are the upper and lower confidence bounds (methods based on [60]). As the reward was never positioned at the end of the entry arm, we considered that learning of the reward position was manifest when the lower confidence bound exceeded 1/4 (black vertical line). **C.** Examples of performance during two individual sessions (monkey K, top; Monkey S, bottom). Each dot represents a correct (blue) or incorrect (red) response (i.e., the monkey did or did not reach the rewarded arm) as a function of trial number (x axis) for the different entries (y axis: starting positions). On the top row, animal K started from entry 1 for the first 36 trials and usually performed incorrectly until the 30^th^ trial, after which the animal performed correctly. On the 37^th^ trial, the animal was asked to reach the reward by starting trials from the other 3 entries. Despite the fact that the animal had never tried to reach the reward from these entries before, the animal performed correctly proving that it used the information acquired on the previous 36 trials to deduce the reward position with respect to the new entries. In the session shown for animal S (bottom), two entry arms were used until trial 60, then the animal was introduced to the remaining two entries to which he performed correctly. When new entries were introduced for all the probe sessions, performances calculated for 5 trials after introduction of the new entries as illustrated by the green box were significantly higher for both monkeys (p = 0.01, Wilcoxon) than for the 5 trials in the beginning of the session as illustrated by the orange box).
(TIFF)

S4 Fig Recording sites. A. Recording sites in monkey S. The far left image shows the location of the recordings on a sagittal section (anterior-posterior (AP) vs dorsal ventral) going through the hippocampus. The next 4 images show the recording sites (yellow dots) plotted on 4 coronal sections slices in millimeters relative to interaural line along the anterior-posterior axis. Each dot corresponds to a recording location. B. Recordings sites in monkey K, corresponding to those for S. (Note the artifact produced by the electrode inserted in the chamber during the imaging on the far right picture.).
(TIFF)

S5 Fig Firing rates. A. Distribution of the mean firing rate for the cells. The majority of the cells displayed a firing rate lower than 5 Hz. B. Distribution of the number of peaks (fields) for the population of cells displaying a spatial selectivity.
(TIFF)

S6 Fig Average neural activity maps. Average neural activity maps, computed for each coding space from the most selective cells (cells for which the IC was very significant, i.e. p < 0.001). Each cell map was normalized to its peak firing rate before computing the population mean. **A**. Position map. Under-represented are areas close to the center and around the first third of the return paths. **B.** Direction map. Inhomogeneities correspond to landmark positions from the center, partly blurred by activity on the return paths. **C.** Point of gaze map. Areas surrounding the landmarks (black rectangles) elicit up to 40% more activity than other areas. **D.** State space map. Center and return paths are inhomogeneously represented, in relation to landmark appearance in the FOV.
(TIFF)

S7 Fig Top view of simulated neural map. Top view of the simulated animal’s trajectory (blue), with simulated spike positions overlaid (red dots). The spike statistics were constrained to create a spatial field (box in space, as seen above).
(TIFF)

S1 Appendix (DOCX)

S2 Appendix (DOCX)

S1 Movie Animal's behavior (infrared view). Monkey S performing the task in his primate chair.
(MP4)

S2 Movie Two-dimensional rendering of the scene, with overlaid point of gaze (task described in subtitles) for monkey S. Video of the task display during trials performed by monkey S with comments explaining the task. Square Dots represent the current point of gaze on the screen (one dot per eye). Monkeys viewed this virtual space in stereo to elicit a sense of immersion; this movie is shown in monocular vision only.
(MP4)

S3 Movie Two-dimensional rendering of the scene, with overlaid point of gaze for monkey K. Video of the task display during trials performed by monkey K.
(MP4)

## Abbreviations

FOV: field of view
IC: information content per spike
VR: virtual reality
