# Supplementary figures and images for: Gaze-informed, task-situated representation of space in primate hippocampus during virtual navigation
Source: PLoS Biol. 2017 Feb 27;15(2):e2001045. doi: 10.1371/journal.pbio.2001045 (PMC5328243; doi:10.1371/journal.pbio.2001045)

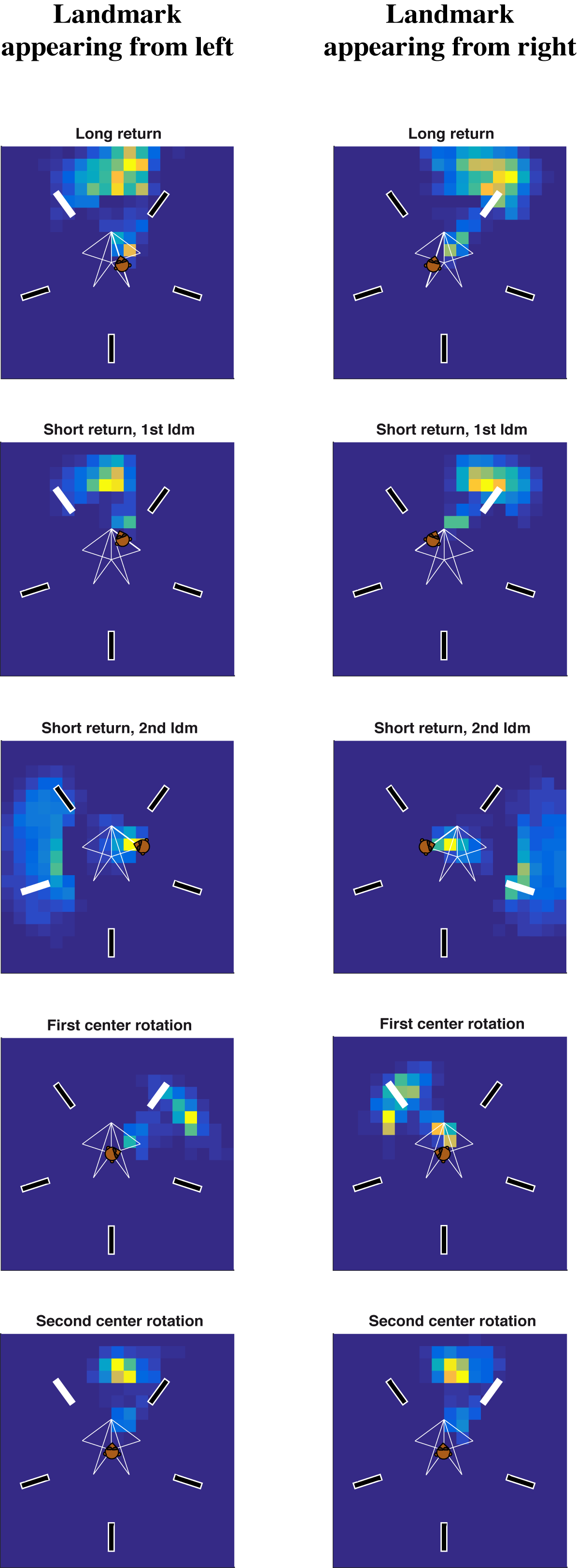

Supplement: S1 Fig — Point of gaze in an allocentric frame representing the maze from the top. The five rectangles represent the five landmarks, with the one highlighted in white, being the landmark (ldm) that appears on the left or on the right of the animal. The inset represents the actual position of the monkey in the paths within the maze (star arms, and passive returns from the north arm end to each of the other 4 arm ends), for short passive return, long passive return, and center rotation. (TIF) [file pbio.2001045.s001.tif]

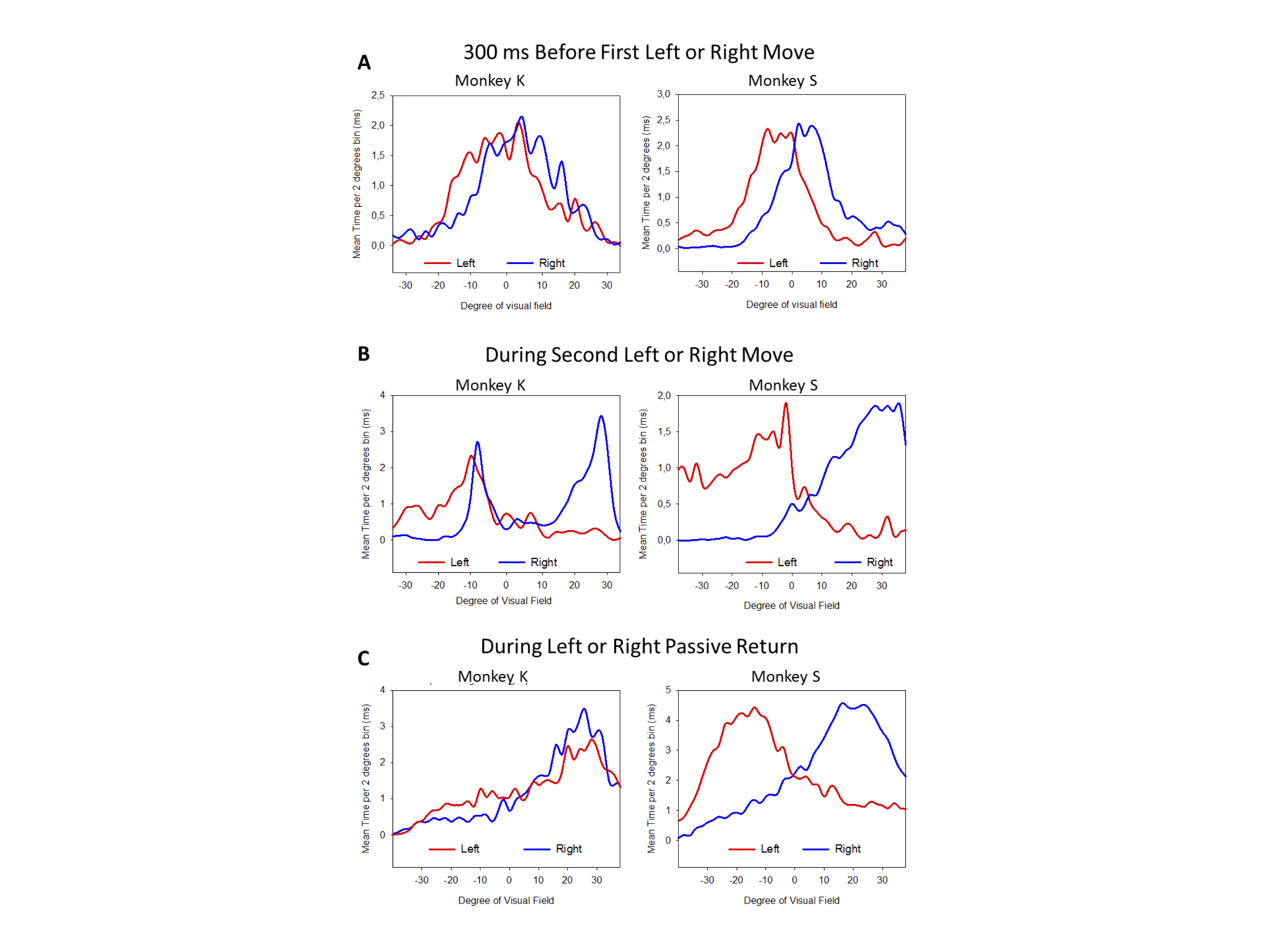

Supplement: S2 Fig — A. Distribution of the mean horizontal eye positions for the 300 ms preceding a turn to the right (in blue) or the left(in r ed) for monkey K (left) and monkey S (right). B. Mean horizontal eye position for the 300 ms preceding the second turn of a series of two turns to the right (in blue) or the left (in red) for monkey K (left) and monkey S (right). C. Mean horizontal eye position during the passive return journey towards the right or the left for monkey K (left) and monkey S (right). (TIFF) [file pbio.2001045.s002.tiff]

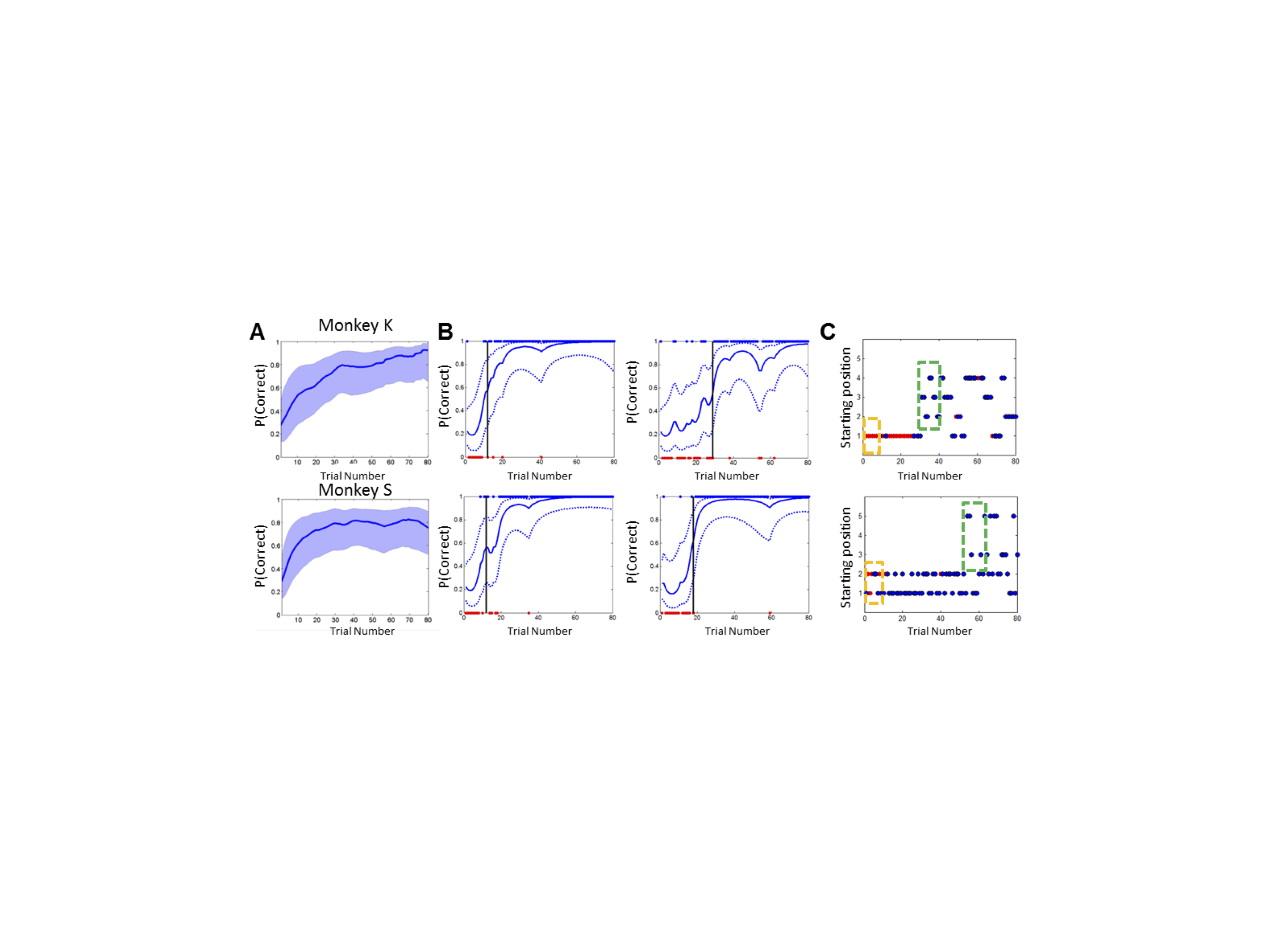

Supplement: S3 Fig — A. Average probability of a correct response over the course of a testing block of 80 trials. B. Learning curves in three representative individual sessions in monkey K (top) and monkey S (bottom). Dots illustrate trial outcome (blue: rewarded, red: non-rewarded). The solid blue line represents the probability of a correct response, and the dotted lines are the upper and lower confidence bounds (methods based on [60]). As the reward was never positioned at the end of the entry arm, we considered that learning of the reward position was manifest when the lower confidence bound exceeded 1/4 (black vertical line). C. Examples of performance during two individual sessions (monkey K, top; Monkey S, bottom). Each dot represents a correct (blue) or incorrect (red) response (i.e., the monkey did or did not reach the rewarded arm) as a function of trial number (x axis) for the different entries (y axis: starting positions). On the top row, animal K started from entry 1 for the first 36 trials and usually performed incorrectly until the 30th trial, after which the animal performed correctly. On the 37th trial, the animal was asked to reach the reward by starting trials from the other 3 entries. Despite the fact that the animal had never tried to reach the reward from these entries before, the animal performed correctly proving that it used the information acquired on the previous 36 trials to deduce the reward position with respect to the new entries. In the session shown for animal S (bottom), two entry arms were used until trial 60, then the animal was introduced to the remaining two entries to which he performed correctly. When new entries were introduced for all the probe sessions, performances calculated for 5 trials after introduction of the new entries as illustrated by the green box were significantly higher for both monkeys (p = 0.01, Wilcoxon) than for the 5 trials in the beginning of the session as illustrated by the orange box). (TIFF) [file pbio.2001045.s003.tiff]

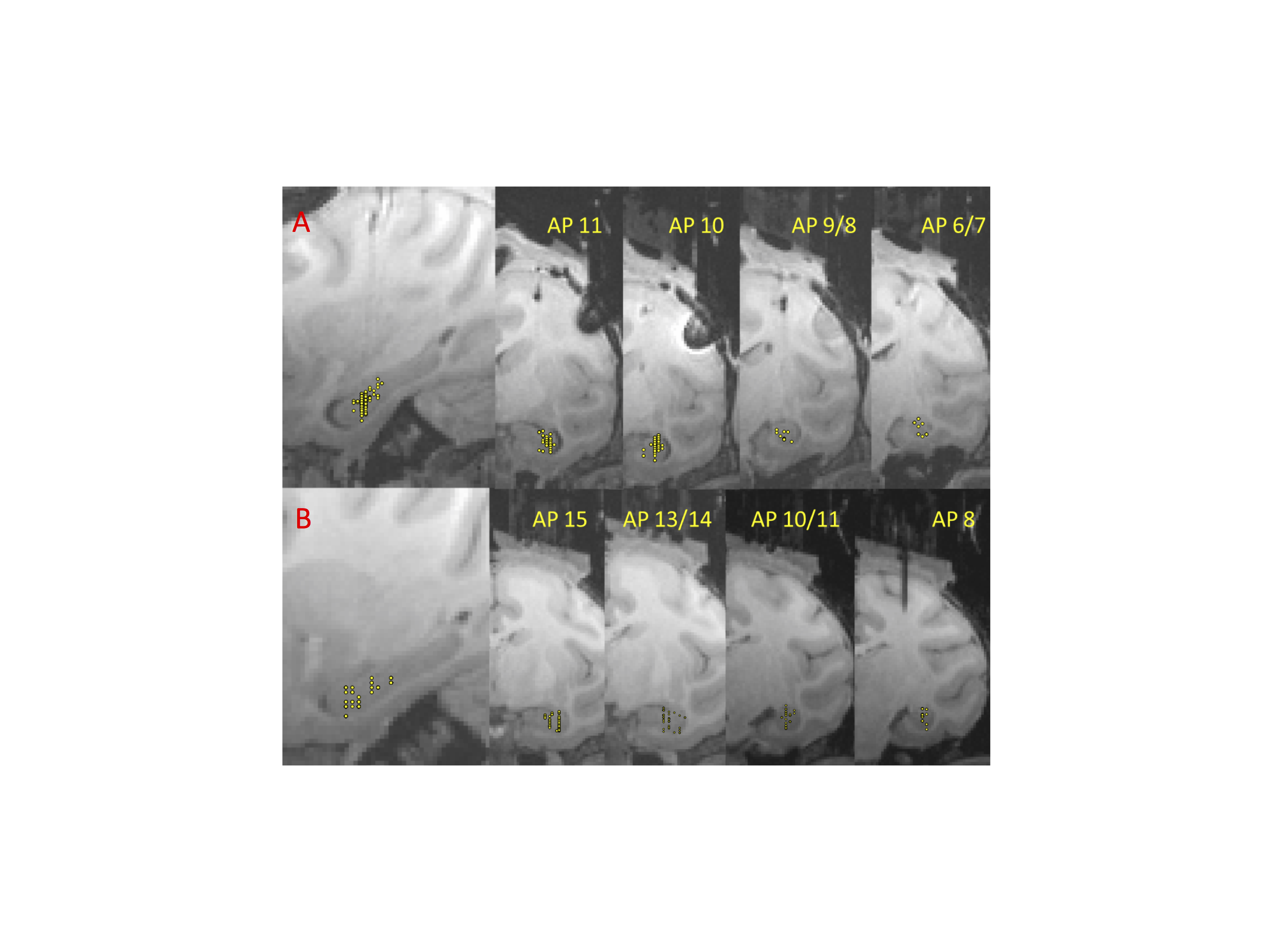

Supplement: S4 Fig — A. Recording sites in monkey S. The far left image shows the location of the recordings on a sagittal section (anterior-posterior (AP) vs dorsal ventral) going through the hippocampus. The next 4 images show the recording sites (yellow dots) plotted on 4 coronal sections slices in millimeters relative to interaural line along the anterior-posterior axis. Each dot corresponds to a recording location. B. Recordings sites in monkey K, corresponding to those for S. (Note the artifact produced by the electrode inserted in the chamber during the imaging on the far right picture.). (TIFF) [file pbio.2001045.s004.tiff]

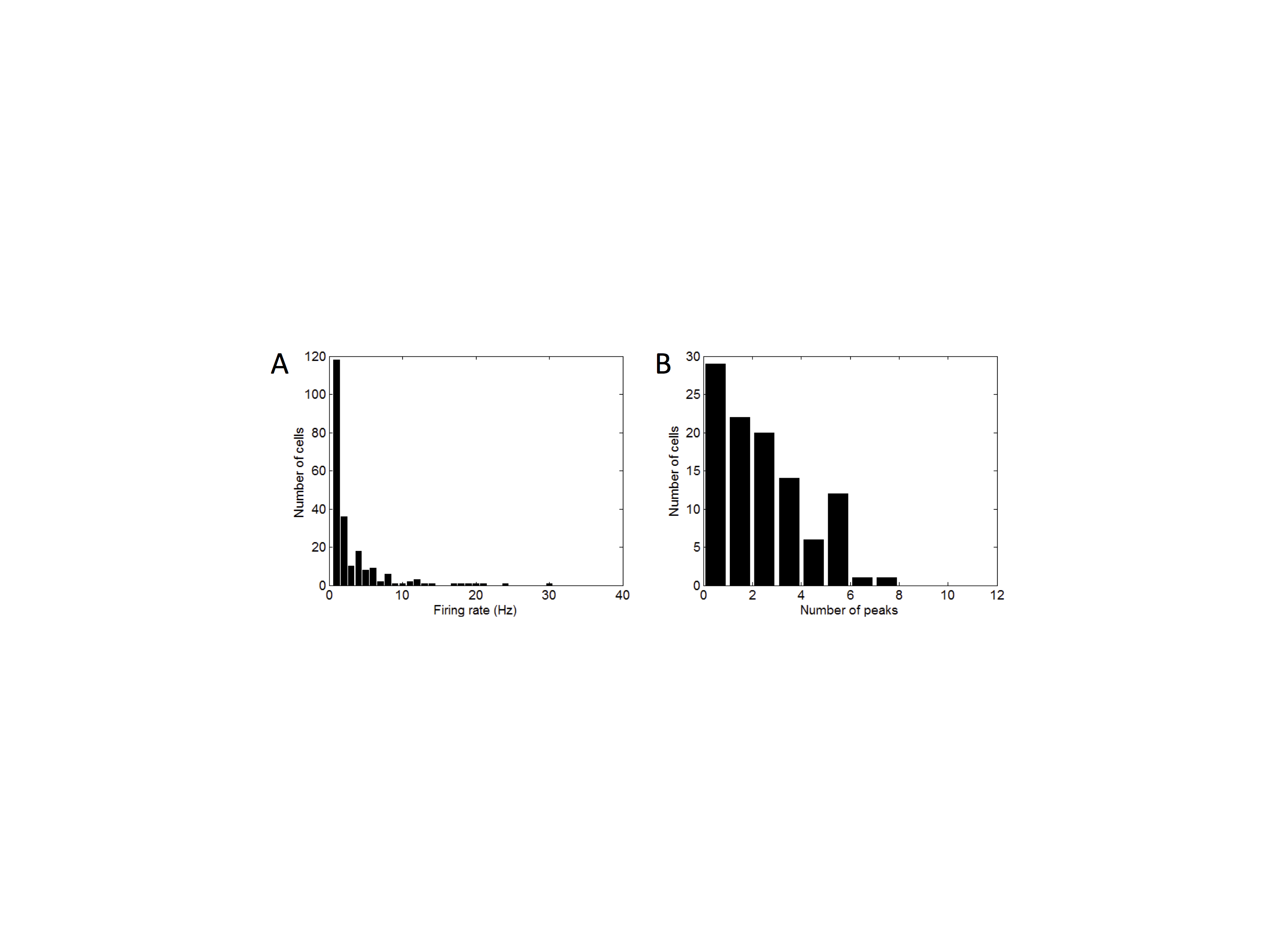

Supplement: S5 Fig — A. Distribution of the mean firing rate for the cells. The majority of the cells displayed a firing rate lower than 5 Hz. B. Distribution of the number of peaks (fields) for the population of cells displaying a spatial selectivity. (TIFF) [file pbio.2001045.s005.tiff]

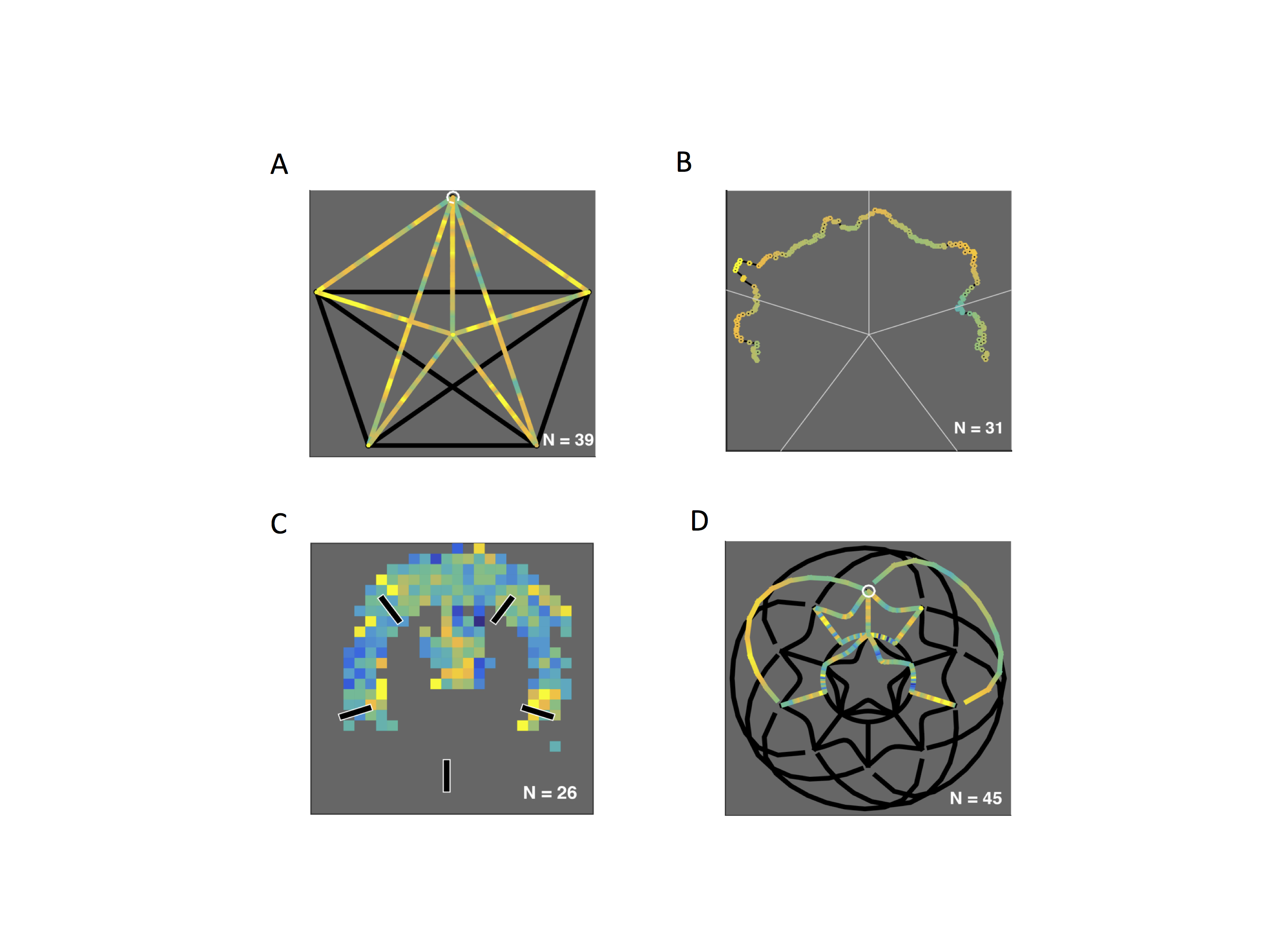

Supplement: S6 Fig — Average neural activity maps, computed for each coding space from the most selective cells (cells for which the IC was very significant, i.e. p < 0.001). Each cell map was normalized to its peak firing rate before computing the population mean. A. Position map. Under-represented are areas close to the center and around the first third of the return paths. B. Direction map. Inhomogeneities correspond to landmark positions from the center, partly blurred by activity on the return paths. C. Point of gaze map. Areas surrounding the landmarks (black rectangles) elicit up to 40% more activity than other areas. D. State space map. Center and return paths are inhomogeneously represented, in relation to landmark appearance in the FOV. (TIFF) [file pbio.2001045.s006.tiff]

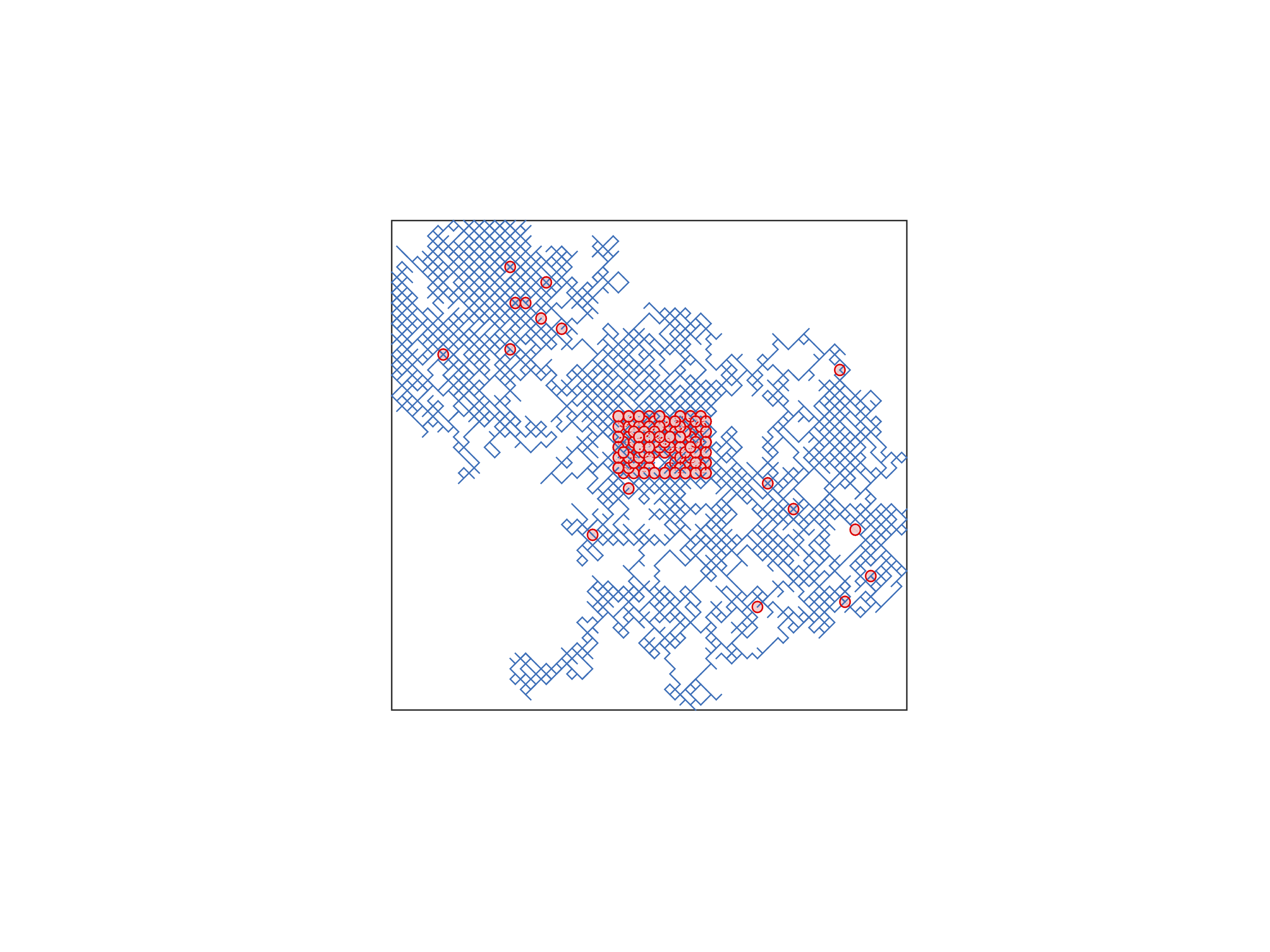

Supplement: S7 Fig — Top view of the simulated animal’s trajectory (blue), with simulated spike positions overlaid (red dots). The spike statistics were constrained to create a spatial field (box in space, as seen above). (TIFF) [file pbio.2001045.s007.tiff]
