# Supplementary material for: Gaze-informed, task-situated representation of space in primate hippocampus during virtual navigation
Source: PLoS Biol. 2017 Feb 27;15(2):e2001045. doi: 10.1371/journal.pbio.2001045 (PMC5328243; doi:10.1371/journal.pbio.2001045)
Supplement: S1 Appendix — (DOCX) [file pbio.2001045.s008.docx]

**A1. Point of gaze in space in an allocentric frame during exploration.**

To determine the targets of the animal’s attention during exploration of the maze, we computed the point of gaze of the animal in an allocentric frame, i.e. the position in space towards which the monkey is directing his gaze, computed for a flattened, top view of the maze (Fig 1D, E, Materials and Methods). In the Fig S1, the density of these points of regard is represented at the moment when a landmark appears on the left (left column) or the right (right column) of the animal’s field of view. We used all behavioral sessions to compute these figures.

The inner schematic represents the current trajectory of the monkey (highlighted in white on the star maze), while the rectangles represent the 5 landmarks. For each trajectory, the landmark that is entering the view is highlighted in white. For the first 3 rows, the animal is travelling paths that place him in front of a new entry (1-3). Row 4 illustrates when the animal is making the first movement of a series of 2. In these cases, the animal samples the upcoming landmark near the reward, with a slight tendency to inspect the right landmark more than the left one. The last row illustrates the point of gaze for the last central rotation towards the reward in trials requiring one movement or a series of two movements. In these cases, the other landmark flanking the reward is already visible and the animal does not need to check the landmark that has just appeared, so he is simply looking towards the goal at the end of the path. The neighboring landmarks alone visually distinguish the end of this rewarded path from the other exits.

**A2. Dynamics of gaze during left or right movements**

To determine whether animals exhibited a gaze behavior compatible with an active exploration of the maze, we analyzed the distribution of the eye position as a function of the up-coming joystick movement behavior. S2A Fig shows the average distribution of the horizontal eye position for each animal 300 ms before they made the first left or right joystick turn. This corresponds to the first choice the animal has to make facing the center of the maze. In both animals, there is a clear shift of eye position that precedes the movement the animal is going to perform. When the animal is going to the left, the eyes positions are skewed towards the left, while the reciprocal phenomenon takes place for movements towards the right. A Wilcoxon test on the distribution of the horizontal position shows that the left and right trials are significantly different. S2B Fig shows the eye position for the 300 ms that precedes the last movement of a series of two movements while the animal is turning. This analysis reveals that on the second turn, the eye position is even more different (p < 0.01, Wilcoxon) between trials where the animal turns to the left and trials where the animal turns to the right.

Finally, we also analyzed the position of the eye, on trials for which the animal’s passive return was towards the right or the left. Fig S2C shows the distribution of the horizontal eye positions for these trials for both monkeys. Monkey S’s gaze strongly accompanied the turn. Monkey K showed a strong bias of looking towards the right during these passive returns. However, this bias was still significantly modulated by the direction of the return in that he consistently fixated the landmark on the right side of the reward position. This behavior revealed what might be a part of a selective memory strategy and reflects individual differences in the monkeys.

**A3. Electrophysiology**

Animals were prepared for chronic recording of single-neuron activity in the right hippocampus. Anesthesia was induced with Zoletil 20 (15 mg/kg) and maintained under isoflurane (2.5%) during positioning of a cilux head-restraint post and recording chamber (Crist Instruments, Damascus, MD). Animals were given atropine (0.25 mg/kg) to prevent excessive salivation. Adequate measures were taken to minimize pain or discomfort. Analgesia was provided by a presurgical buprenorphine injection (0.2 mg/kg). The position of the recording chamber for each animal was determined using stereotaxic coordinates derived from pre-surgical anatomical magnetic resonance images (MRI, 0.6 mm isometric (1.5 Tesla), to have access to the right hippocampus and right parietal cortex structure simultaneously. Postsurgical MR images were used to finely monitor recording locations during each experiment (S4 Text and S4 Fig). The MR images were taken with an electrode of known depth inserted in the middle section of the chamber (see supplementary data 4). During recordings, this reference was used to measure positions of the electrode tip in the brain. To locate the hippocampus, we used physiological neural signatures to identify structures above our regions of interest and passing through the ventricle bordering the anterior portions of the hippocampus. To perform the recordings, we used either single contact tungsten electrode (1–2 MΩ; Frederick Haer Company, Bowdoinham, ME), or 16 contact U-probes spaced by 300µm (Plexon Inc, Dallas, USA). Electrodes were lowered slowly until they reached the hippocampus, and cells could be identified. Once there was recorded activity, we started calibrating the monkey’s eye signals, and then started the behavioral task.

**A4. Average activity maps**

Map inhomogeneity was quantified with Moran’s *I*, and statistically tested with permutation tests, as documented in the table below. Moran’s *I* index essentially characterizes spatial autocorrelation (-1 ≤ *I* ≤ +1). For a random map, *I* ~ 0, and *I* increases towards +1 for a clustered map (*I* = –1 for a perfectly dispersed map like a checkerboard).

|  | Position | Direction | Point of gaze | State space |
| --- | --- | --- | --- | --- |
| Moran’s *I* | 0.34 (p = 0.02) | 0.38 (p = 0.002) | 0.24 (p = 0.01) | 0.56 (p = 0.002) |

Thus all average maps show a statistically significant degree of clustering. Looking at their spatial characteristics, this clustering seems often related to landmark appearance and/or fixation.

**A5. A space with higher dimensionality does not necessarily have higher information content.**

Though information content is evaluated in exactly the same number of bins in all spaces, the State space includes more dimensions in its construction. Though the situation bears no resemblance to model fitting, in which parameter number has an importance, one may wonder whether the added complexity in the definition of the State space is enough to explain its higher information content (IC). To show that a more complex space does not necessarily have a higher IC, we ran the following simulation.

A virtual animal navigated a three-dimensional 100x100x9 cm box environment (3D random walk with spatial resolution 1 cm, illustrated by blue lines on S7 Fig, box seen from above). In this space, we supposed that a neuron has a preferred zone, in which it fires at 25 Hz (Poisson firing statistics). Outside this field, it fires at 0.1 Hz. On S7 Fig, spikes are represented as red dots at the spatial position they occurred. We studied two cases: either the place field extends fully across the vertical (a 18x12x9 cm box in which the firing does not depend on Z, as shown above), or this place field is restricted to a subspace of heights (a 18x36x3 cm box, Z > 6 cm; note that fields have same volume). For each virtual trial, we ran our information content estimation procedure, comparing two spaces: a 2D space, or a 3D space.

Since the 3D space is defined with one more parameter than the 2D space, it is an analog of the State space, which involves more dimensions than the Position space. As in the procedure that we used on actual data, we took care to equalize bin numbers across spaces in our information content estimation (196 bins, either a 14x14 square, or a 7x7x4 box) and normalize the IC on the surrogate, spike shifted datasets. This measure of IC was repeated in 200 ‘trials’ of 3 minutes.

When firing was independent of Z, the IC for the 2D space was higher than for the 3D space (average IC_2D_ = 1.41 vs. IC_3D_ = 0.95, paired Wilcoxon p < 0.0001). When firing was Z-dependent, the converse was true (IC_2D_ = 0.77 vs. IC_3D_ = 1.34, paired Wilcoxon p < 0.0001). Thus the more complex space does not necessarily have higher IC. The space with highest IC is simply the one that better reflects the actual organization of the firing fields.
