# Supplementary material for: Gaze-informed, task-situated representation of space in primate hippocampus during virtual navigation
Source: PLoS Biol. 2017 Feb 27;15(2):e2001045. doi: 10.1371/journal.pbio.2001045 (PMC5328243; doi:10.1371/journal.pbio.2001045)
Supplement: S2 Appendix — (DOCX) [file pbio.2001045.s009.docx]

**This appendix describes the cells included in Fig 1-7.**

Underlying data can be found at [http://dx.doi.org/10.6080/K0R49NQV.](http://dx.doi.org/10.6080/K0R49NQV.%E2%80%9D)

**Data for Fig 1.**

Heat maps of Eye positions (Fig 1D) and allocentric point of view (1E) are calcutated with the X,Y eye data provided in the supplementary data sn20110802_e1_HPC_c2_segcln.

**Data for Fig 2.**

Each data set corresponds to one of the 8 cells shown in Fig 2 and is provided in the supplementary data. Each data set contains XY coordinates and camera orientation in the virtual world, timestamps of the action potentials. state-space coordinates, x and y eye position coordinates for the right and left eye. We provide a Matlab script that plots the position map, the head direction map, the state-space map, the rasterhistogram aligned on each beginning of a trial, and the behavioural learning curve.

Cell 1 : sn20130215_HPC_e1_c2_segcln

Cell 2: sn20130215_HPC_e2_c2_segcln

Cell 3: sn20110802_e1_HPC_c2_segcln

Cell 4: ke20130315_HPC_e11_c1_segcln

Cell 5: sn20121206_HPC_e1_c1_segcln

Cell 6: ke20140228_HPC_c1_segcln

Cell 7: sn20121218_HPC_e1_c1_segcln

Cell 8: ke20131122_HPC_e1_c2_segcln

**Data for Fig 3.**

Data is contained within the figure itself.

**Data for Fig 4.**

Files are provided in the supplementary data.

Figure 4C : sn20120926_HPC_e1_c2
Figure 4D : ke20130301_HPC_e1_c1

**Data for Fig 5.**

Files are provided in the supplementary data.

5A: sn20110624_e1_HPC_c2_segcln

5B: sn20110624_e1_HPC_c1_segcln

**Data for Fig 6.**

Files are provided in the supplementary data.

A: sn20130215_HPC_e2_c2_segcln (cell 2 in Fig 1)

B: sn20130215_HPC_e1_c2_segcln (cell 1 in Fig 1)

C: sn20121218_HPC_e1_c1_segcln (cell 7 in Fig 1)

**Data for Fig 7.**

Files are provided in the supplementary data.

A and B: sn20121206_HPC_e4_c2_segcln

C and D: sn20130215_HPC_e2_c1_segcln

-----

We have provided a matlab master script that can be ran on any of the files and that will plot for each cell:

A position map, a head direction map, a state space map, a raster histogram aligned on the beginning of each trial, and the learning curve for the behavior.

This master script function is entitled ‘plot_multiple_ref_frames’ and should be ran in matlab as follows :

plot_multiple_ref_frames(‘filename’, ‘newallo’) given that the path has been set to access the functions in the folder.

Each file starting by ‘ke’ or ‘sn’ is a matlab datafile object containing the following fields:

file.elec: times stamps of the action potentials of the neuron

file.right_eye: hor and ver eye position at a resolution of 250hz

file.left_eye: hor and ver eye position at a resolution of 250hz

file.eye_times: corresponds to the absolute time for the eye positions

file.newallo.codematrix: a matrix with the codes of the events in the trials. Each row corresponds to a single trial.

file.newallo.codematrix: a matrix with the timestamps of codes of the events in the trials. Each row corresponds to a single trial and corresponds to the events in the codematrix.

file.newallo.learning_curves: the probability of a correct response (middle column), upper and lower bounds (1^st^ and 3^rd^ columns).

file.camera_data: columns 1-4: timestamp of the each coordinates shown in the other columns, x position, y position, orientation of the camera.

Columns 5 to 12: state-space coordinates (see script for how to plot the coordinates)

file. reclocation: AP,ML and DV recording location with respect to interaural line.

Correspondence of the event codes in the field file.newallo.codematrix:

Codes 40-44: identity of the starting arm

21: start of the trial

3: joystick move forward

22: arrival at choice point

2: movement to the left

1: movement to the right

50:54: identity of the chosen arm

11: correct trial reward given

12: error trial

23: end of the trial
